# Supplementary material for: Recurrent introgression and geographical stratification shape Saccharomyces cerevisiae in the Neotropics
Source: Nat Commun. 2026 Feb 21;17:3024. doi: 10.1038/s41467-026-69138-0 (PMC13035892; doi:10.1038/s41467-026-69138-0)
Supplement: Supplementary file 1 — Supplementary Information [file 41467_2026_69138_MOESM1_ESM.pdf]

Supplementary Information

Recurrent introgression and geographical stratification shape *Saccharomyces cerevisiae* in the Neotropics

J. Abraham Avelar-Rivas, Iván Sedeño, Luis F. García-Ortega, Jose A. Urban Aragon, Claudio López-Gallegos, Xitlali Aguirre-Dugua, Eugenio Mancera*, Alexander DeLuna*, Lucia Morales*

**Correspondence to:*

- Eugenio Mancera* (eugenio.mancera@cinvestav.mx),
- Alexander DeLuna* (alexander.deluna@cinvestav.mx), and
- Lucia Morales* (lmorales@liigh.unam.mx)

This PDF file includes:

Supplementary Figure Legends 1-9

Supplementary Figures 1-9

References

SUPPLEMENTARY FIGURE LEGENDS

Supplementary Figure 1. Origin of the 216 sequenced strains from sampling efforts conducted across the country between the years 1988 and 2021[1–9]. The shape denotes the sampling reference, and the color indicates the year in which isolates were collected.

Supplementary Figure 2. The Neotropical cluster remains coherent regardless of sampling depth or the presence of introgressions. **a** Multidimensional Scaling (MDS) analysis of a downsampled dataset including up to five strains per clade with introgressions. The MDS plot was generated using SNPs called from alignments to the *S. cerevisiae* reference, which retained SNPs from introgressed regions. **b** MDS plot generated using SNPs called from alignments to a concatenated reference keeping only SNPs found in the *S. cerevisiae* nuclear subgenome, thereby excluding SNPs from introgressions. **c,** Maximum-likelihood phylogeny with a set of 486 genomes, including SNVs within introgressions (1,177,709 SNPs). **d,** Maximum-likelihood phylogeny with the same set of 486 strains, but excluding introgressions (1,002,285 SNPs). **e** Maximum-likelihood phylogeny of 332 strains as described in the main text Figure 1B. **f** Genetic differentiation among Neotropical groups, using weighted *Fst*. **g-h** Maximum-likelihood phylogenies inferred from a downsampled set of genomes (up to five strains per clade), including (**g**) or excluding (**h**) introgressed regions. All phylogenies were made with IQTree using ascertainment bias correction, with aLRT support indicated for key nodes. Colored dots at the tip of the branches show the origins of *S. cerevisiae* strains from the Neotropical cluster: Mexican Agave 1 (purple), Mexican Agave 2 (blue), French Guiana (orange), Tequila Distillery (yellow), Wild Brazil 3 (carmine), South American Mix 2 (pink); Wine, Alpechin, Mosaic Region and Taiwanese strains are labeled for reference.

Supplementary Figure 3. Neotropical strains grouped in clades with elevated SNV counts relative to worldwide diversity. **a** Box plots show the 25th and 75th percentiles, with red horizontal lines indicating the median SNV counts for each clade across strains representing the global diversity (n= number of strains per clade). Neotropical clades are shown on the far left, and the remaining clades are ordered by the median of SNV count (red). Variants from the newly sequenced strains were merged with the VCF from Peter *et al.*, 2018[10], and variant counts were computed using bcftools 1.9. **b** Median identity by sequence between clades, calculated from a VCF containing SNVs from the 487 genomes used for the complete phylogenetic reconstruction.

Supplementary Figure 4. Population structure analysis with ADMIXTURE. **a** Cross-validation error analysis across K values, with each dot representing one of ten replicate runs. Cyan and magenta lines indicate the mean and the median cross-validation error, respectively. **b** ADMIXTURE population structure analysis for the models with the lowest cross-validation error (K= 3-26). South American Mix clades (Tellini et al. 2024)[11] include the following: SAM1 is formed by strains of B1 lineage of Barbosa et al. (2016)[12] and Ecuadorean strains from Peter et al. (2018)[10], SAM2 is formed by the WB3 lineage of Barbosa et al. (2016)[12] and different unassigned strains from the Americas from Peter et al. (2018)[10] , and SAM3 includes lineage B4 of Barbosa et al. (2016)[12], Cachaca strains of Barbosa et al. (2018)[13] and a Brazil spirit strain from Gallone et al. (2016)[14].

Supplementary Figure 5. Biocultural regions of agave fermentation used for agave spirit production show a gradient in genetic diversity. **a** Box plots show the 25th and 75th percentiles, with vertical lines indicating median nucleotide diversity (π) calculated in 10-kb windows. **b** Genome-wide heterozygosity. The dashed line indicates the threshold used to define homozygous strains in Peter *et al*., (2018)[10]. **c** Map showing regions in different colors, each defined as in Gallegos-Casillas *et al*., (2024)[1].

Supplementary Figure 6. Genetic diversity parameters across clades from the Neotropical cluster. Box plots show the 25th and 75th percentiles, with horizontal lines indicating the median values of nucleotide diversity (π, **a**), Tajima's D (**b**), and Heterozygosity (**c**). Whiskers extend to the minimum and maximum values within 1.5x the interquartile range. More extreme values are not displayed explicitly. Matrices on the right display the *p*-values from two-sided Mann-Witney U pairwise tests, as indicated in the color code. We considered clades with more than ten available strains (MA1, n=20; MA2, n=178; Tequila, n=15; FG, n=29; Wine1, n=23; Alpechin, n=12; Wine2, n=11). For genetic diversity and Tajima's D analyses, box plots were generated using 10-kb windows, with the following numbers of windows per clade: MA1, n=1,135; MA2, n=1,145; Tequila, n=1,144; FG, n=1,139; Wine1, n=1,140; Alpechin, n=1,134; and Wine2, n=1,137.

Supplementary Figure 7. Comparison of the introgression detection method used in this study with alternative approaches. **a** Venn diagram showing the number of genes identified as introgressed across 46 shared strains using three different methods: this study; Tellini et al., 2024[11]; Peter et al, 2018[10]. **b** Venn diagram showing the number of genes identified as introgressed across 3 shared strains using three different identification methods: this study; Tellini et al., 2024[11]; Barbosa et al, 2016[10].

Supplementary Figure 8. Introgression block size across clades of the Neotropical cluster. Strains with more genes per block tend to also show a higher number of introgression blocks. WB3, Wild Brazil 3; FG, French Guiana; SAM2, South American Mix 2.

Supplementary Figure 9. Cladogram of MA1 alongside a strain-level, presence-absence matrix of introgressed genes. Phylogenetic relationships were extracted from the phylogeny in Fig. 1b. The color of each gene reflects the ratio of read counts between the *S. cerevisiae* and *S. paradoxus* alleles. Introgressions with ratios between 0.25 and 4 are classified as heterozygous (red), whereas lower ratios are classified as homozygous or hemizygous. For FG and MA2, twenty randomly selected strains are shown as references.

Supplementary Figure 1

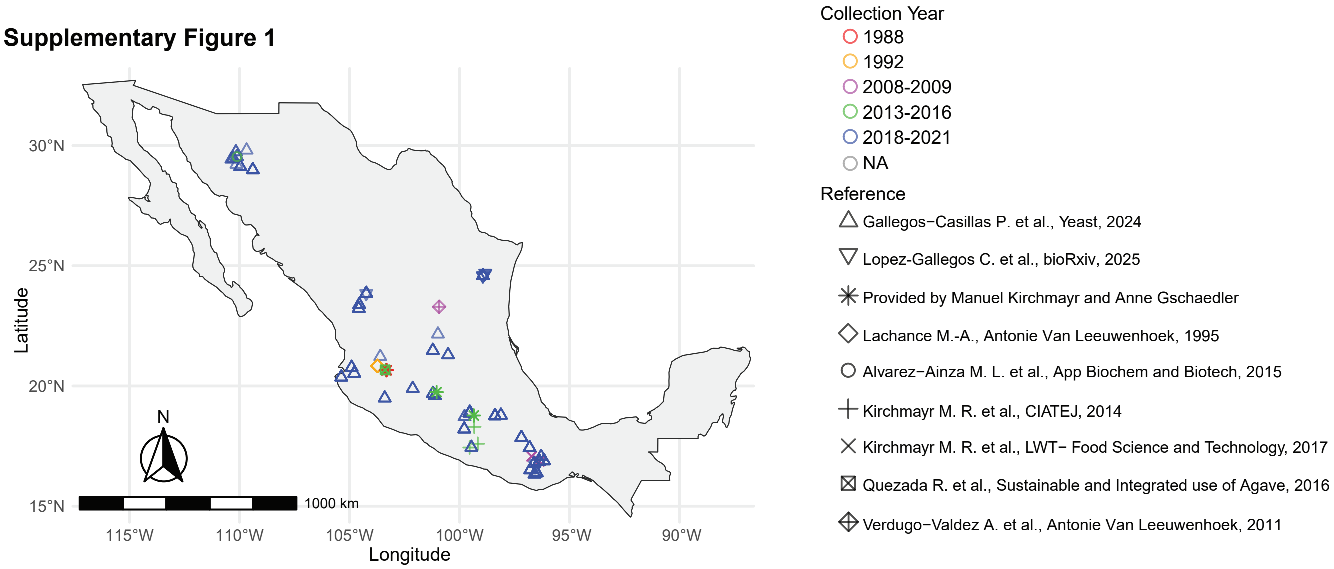

Supplementary Figure 2

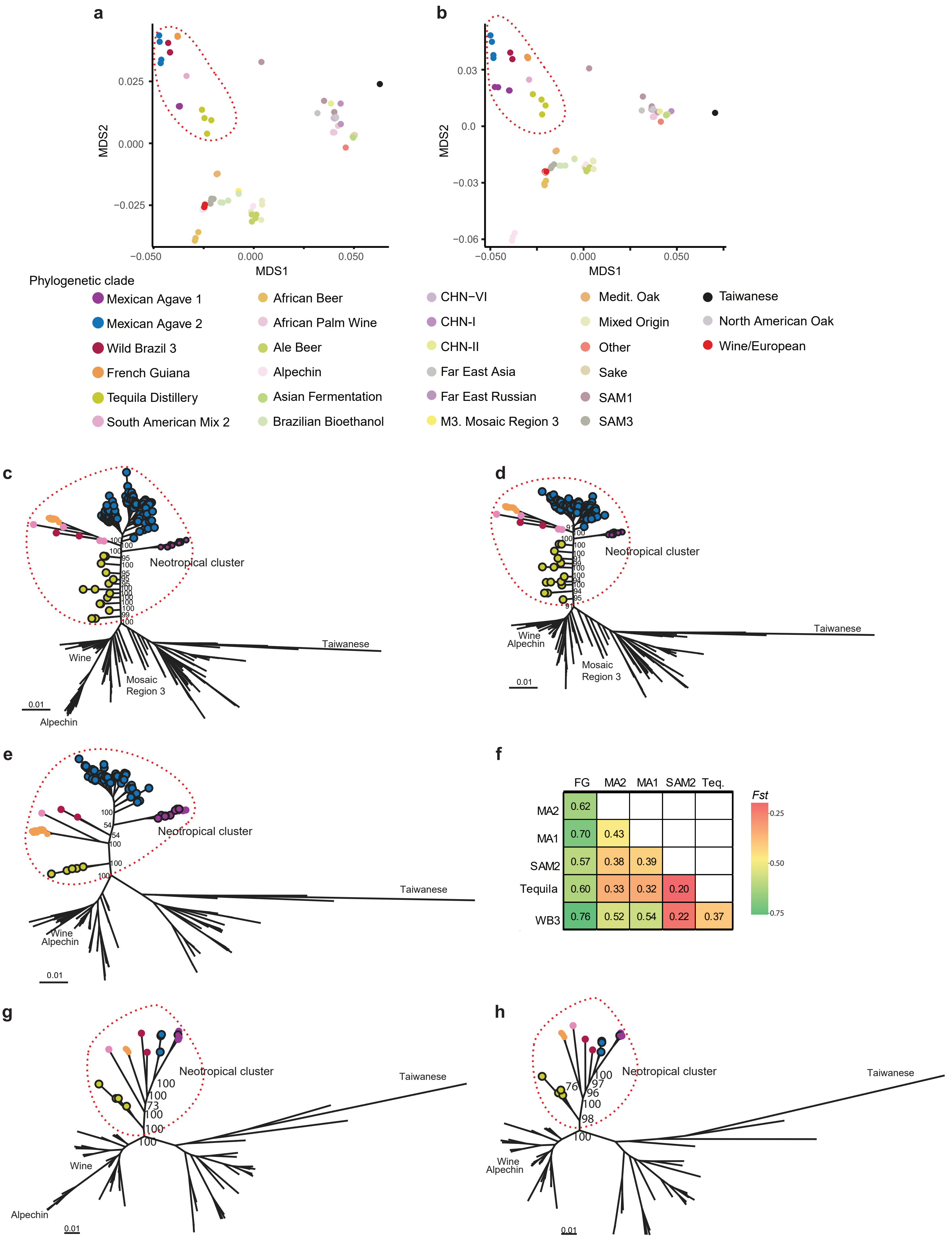

Supplementary Figure 3

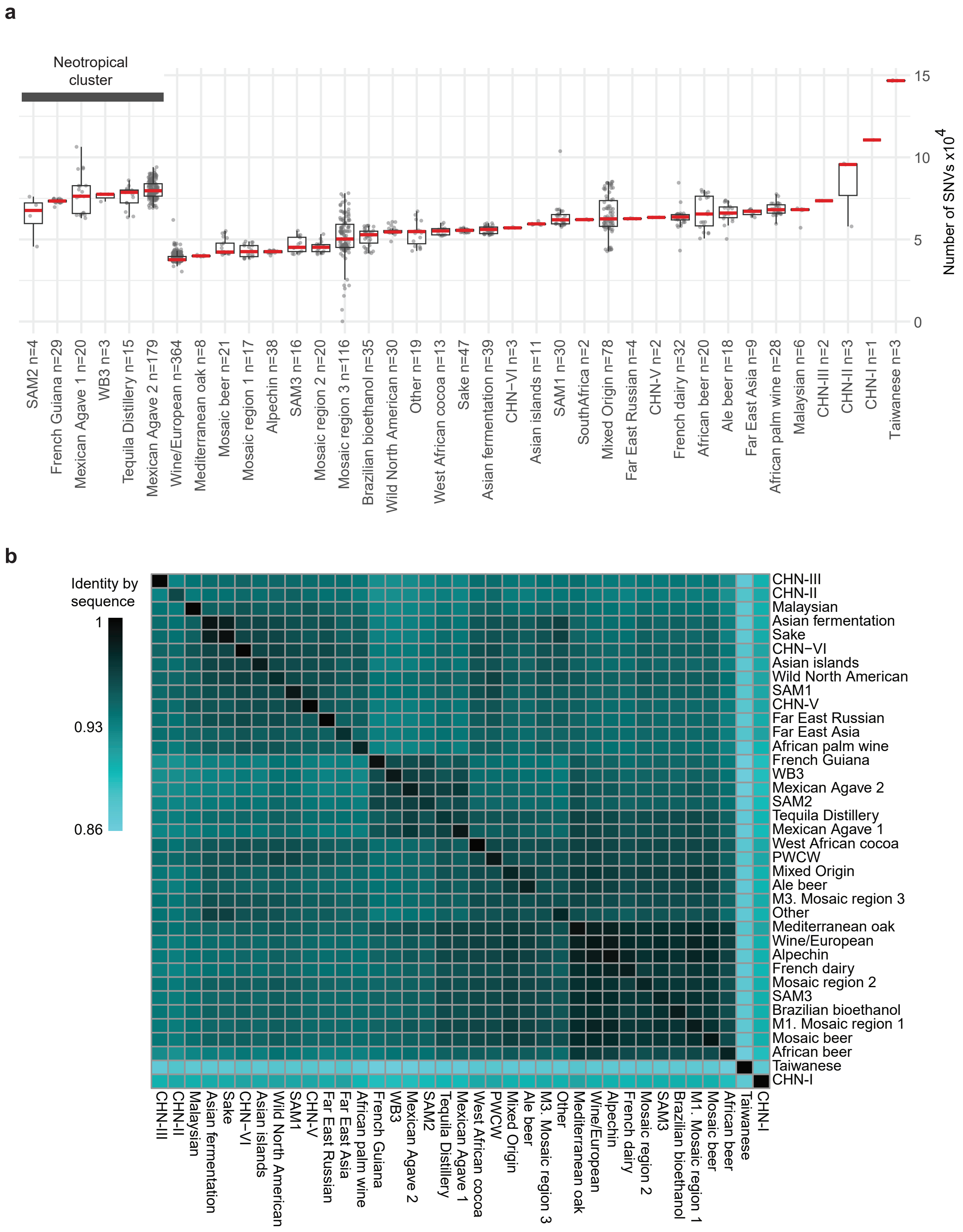

Supplementary Figure 4

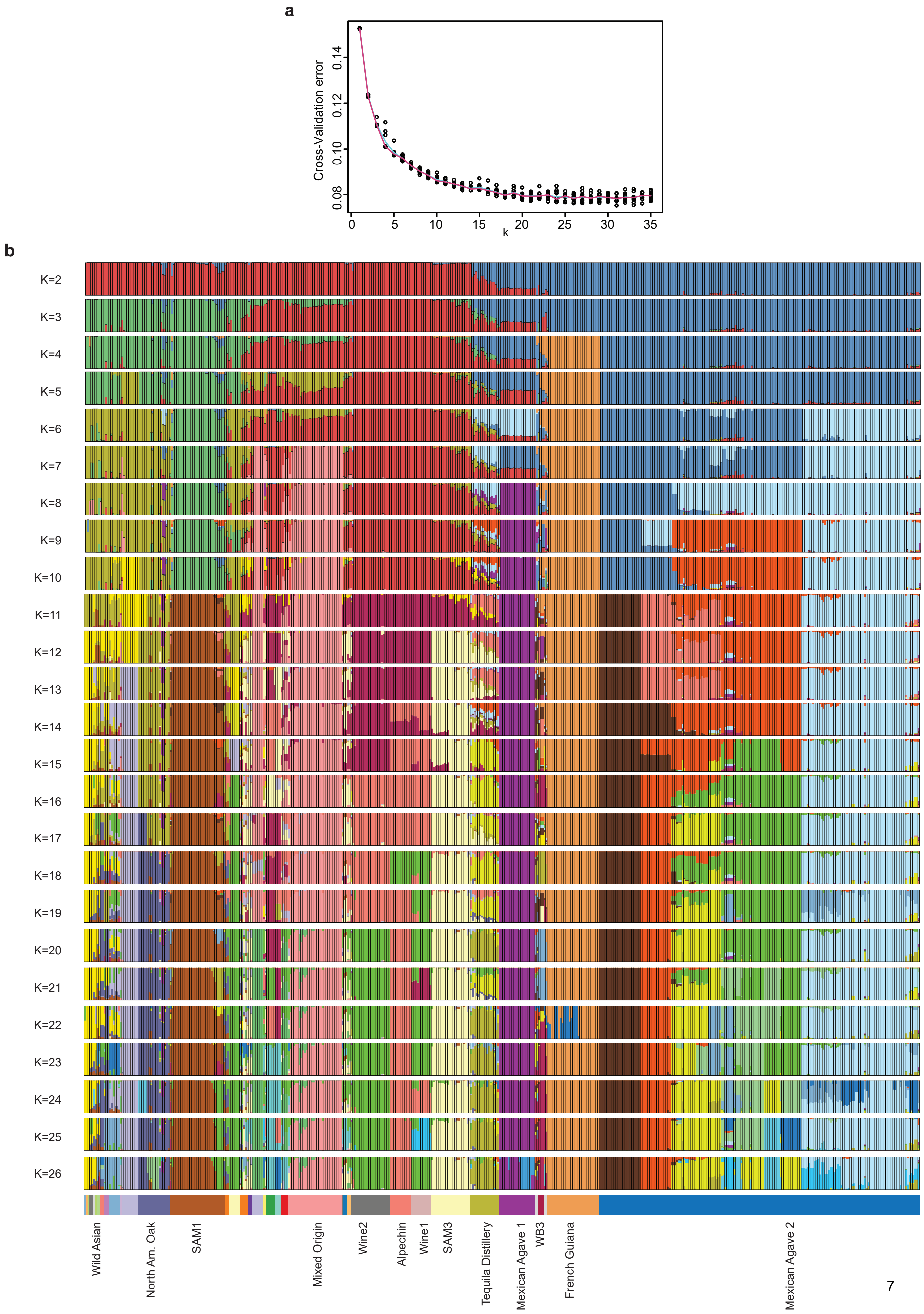

Supplementary Figure 5

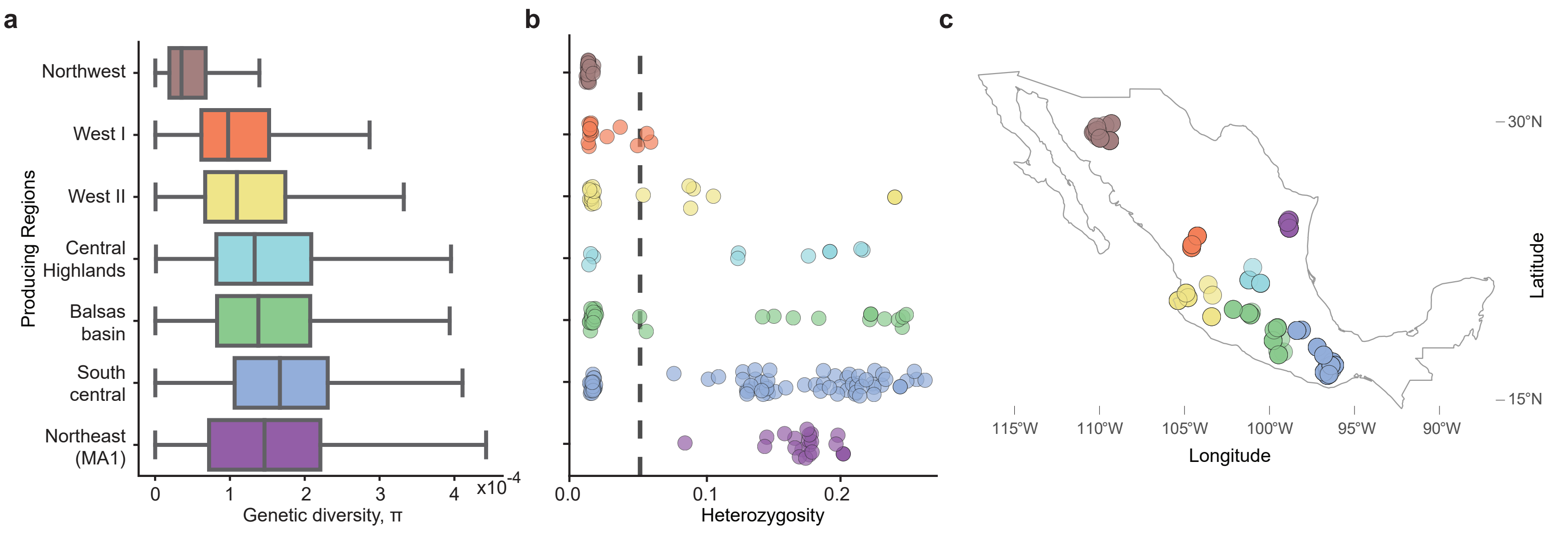

Supplementary Figure 6

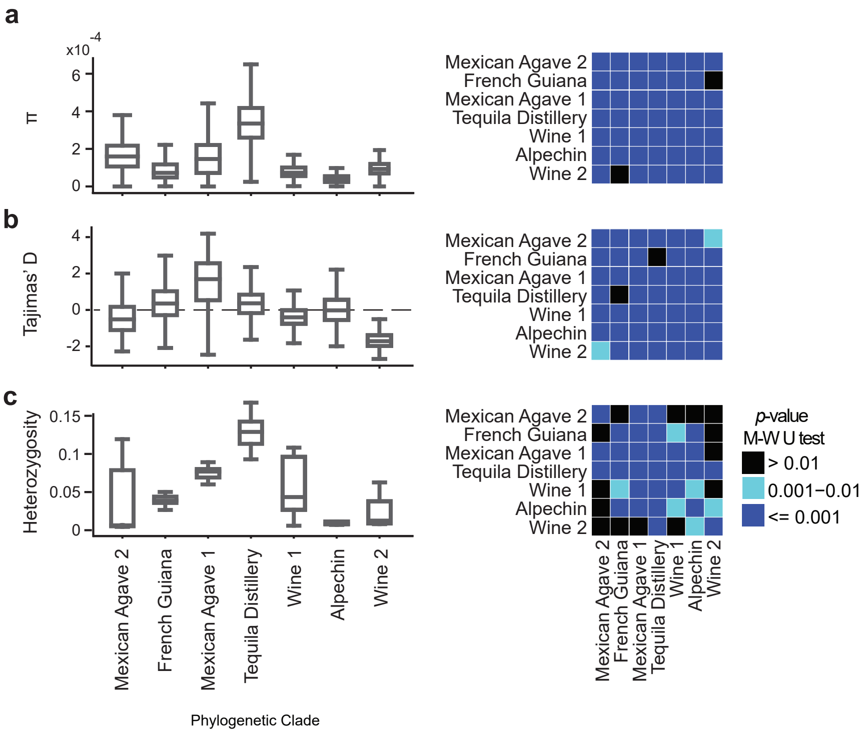

Supplementary Figure 7

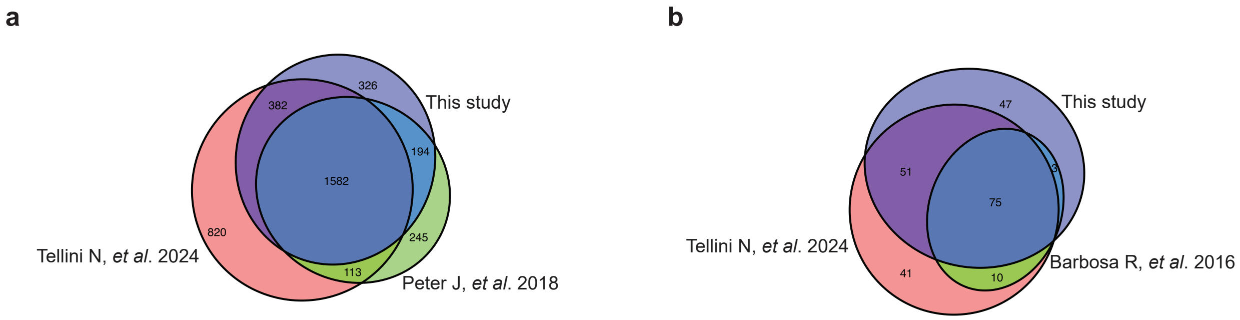

Supplementary Figure 8

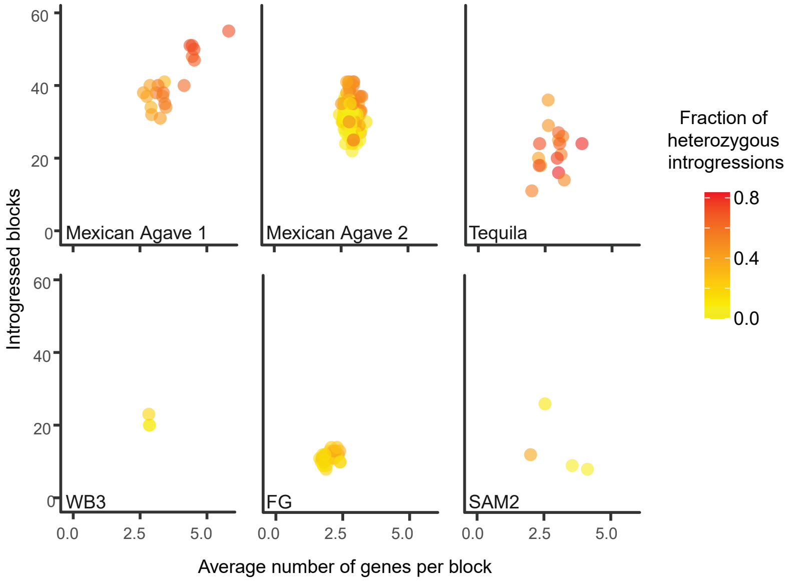

Supplementary Figure 9

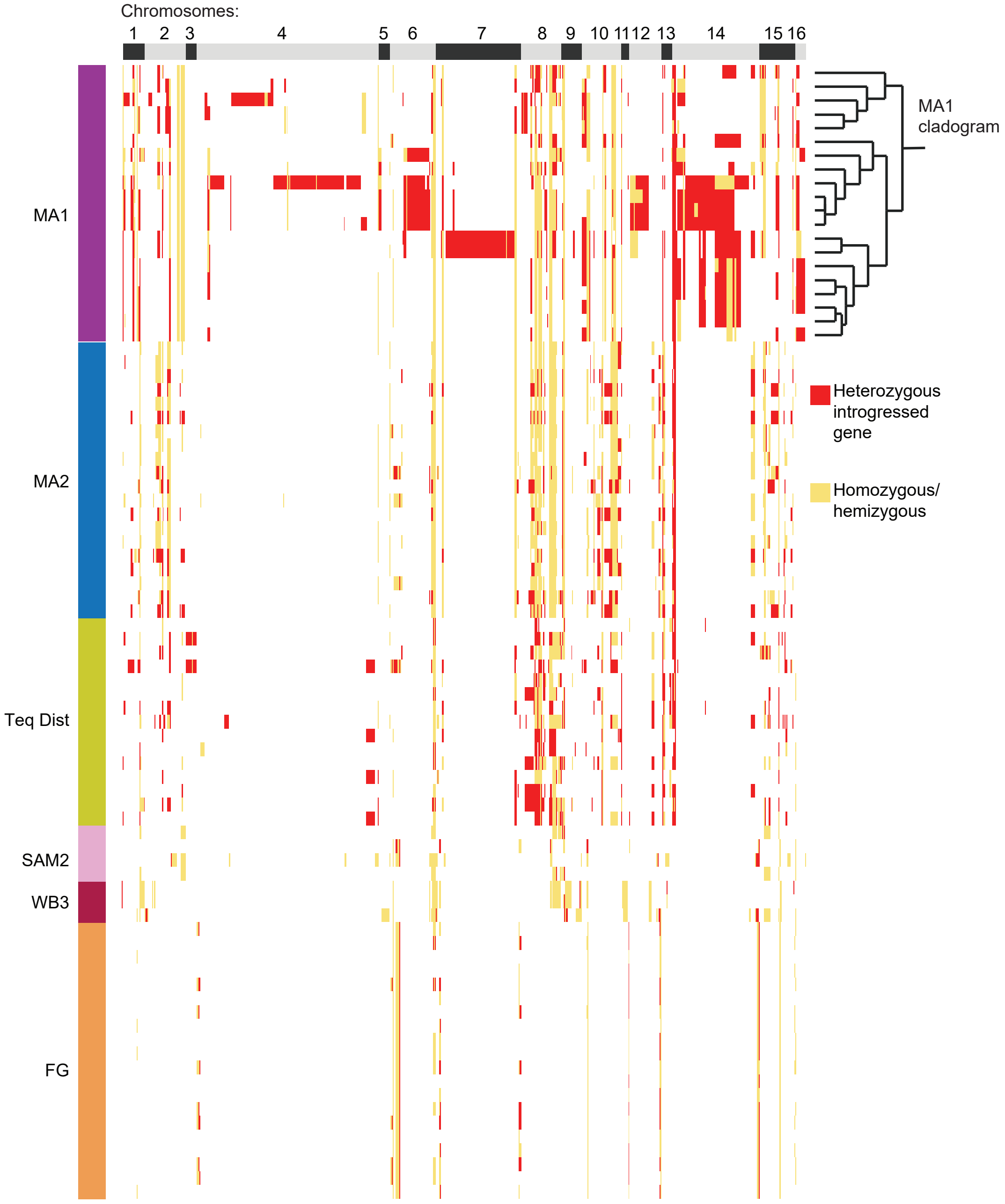

REFERENCES

1. Gallegos-Casillas, P. *et al.* Yeast diversity in open agave fermentations across Mexico. *Yeast* **41**, 35–51 (2024).
2. López-Gallegos, C. *et al.* Ecological divergence of sympatric *Saccharomyces* species across wild and fermentative environments in the neotropics. 2025.05.31.656962 Preprint at https://doi.org/10.1101/2025.05.31.656962 (2025).
3. Lachance, M.-A. Yeast communities in a natural tequila fermentation. *Antonie van Leeuwenhoek* **68**, 151–160 (1995).
4. Verdugo Valdez, A. *et al.* Yeast communities associated with artisanal mezcal fermentations from *Agave salmiana*. *Antonie van Leeuwenhoek* **100**, 497–506 (2011).
5. Kirchmayr, M. R. *et al.* Impact of environmental conditions and process modifications on microbial diversity, fermentation efficiency and chemical profile during the fermentation of *Mezcal* in Oaxaca. *LWT - Food Science and Technology* **79**, 160–169 (2017).
6. Padilla-Camberos, E., Pinal-Zuazo, L. & Alvarez de la Cuadra Jacob, J. Catálogo de la colección de cultivos microbianos. (1994).
7. Kirchmayr, M. R. *et al. Manual para la estandarización de los procesos de producción del mezcal guerrerense*. vol. 1 (Centro de Investigación y Asistencia en Tecnología y Diseño del Estado de Jalisco A.C., Guadalajara, Jalisco. México, 2014).
8. Quezada, R., Gschaedler, A. & Kirchmayr, M. Characterization of microbial population dynamics associated with different juices of *Agave tequilana*. in *Sustainable and Integrated use of Agave* vol. 2016 2016 (2016, Zapopan, Jalisco, Mexico, 2016).
9. Álvarez-Ainza, M. L., Zamora-Quiñonez, K. A., Moreno-Ibarra, G. M. & Acedo-Félix, E. Genomic Diversity of *Saccharomyces cerevisiae* Yeasts Associated with Alcoholic Fermentation of Bacanora Produced by Artisanal Methods. *Appl Biochem Biotechnol* **175**, 2668–2676 (2015).
10. Peter, J. *et al.* Genome evolution across 1,011 *Saccharomyces cerevisiae* isolates. *Nature* **556**, 339–344 (2018).
11. Tellini, N. *et al.* Ancient and recent origins of shared polymorphisms in yeast. *Nat Ecol Evol* **8**, 761–776 (2024).
12. Barbosa, R. *et al.* Evidence of Natural Hybridization in Brazilian Wild Lineages of *Saccharomyces cerevisiae*. *Genome Biol Evol* **8**, 317–329 (2016).
13. Barbosa, R. *et al.* Multiple Rounds of Artificial Selection Promote Microbe Secondary Domestication—The Case of Cachaça Yeasts. *Genome Biology and Evolution* **10**, 1939–1955 (2018).
14. Gallone, B. *et al.* Domestication and Divergence of *Saccharomyces cerevisiae* Beer Yeasts. *Cell* **166**, 1397-1410.e16 (2016).
