# Supplementary material for: Recurrent introgression and geographical stratification shape Saccharomyces cerevisiae in the Neotropics
Source: Nat Commun. 2026 Feb 21;17:3024. doi: 10.1038/s41467-026-69138-0 (PMC13035892; doi:10.1038/s41467-026-69138-0)
Supplement: Supplementary file 3 — Reporting Summary [file 41467_2026_69138_MOESM3_ESM.pdf]

nature portfolio

Corresponding author(s): Eugenio Mancera, Alexander DeLuna, Lucía Morales

Last updated by author(s): Dec 12, 2025

Reporting Summary

Nature Portfolio wishes to improve the reproducibility of the work that we publish. This form provides structure for consistency and transparency in reporting. For further information on Nature Portfolio policies, see our Editorial Policies and the Editorial Policy Checklist.

Statistics

For all statistical analyses, confirm that the following items are present in the figure legend, table legend, main text, or Methods section.

n/a	Confirmed	
[ ]	[x]	The exact sample size (*n*) for each experimental group/condition, given as a discrete number and unit of measurement
[x]	[ ]	A statement on whether measurements were taken from distinct samples or whether the same sample was measured repeatedly
[ ]	[x]	The statistical test(s) used AND whether they are one- or two-sided<br>*Only common tests should be described solely by name; describe more complex techniques in the Methods section.*
[x]	[ ]	A description of all covariates tested
[x]	[ ]	A description of any assumptions or corrections, such as tests of normality and adjustment for multiple comparisons
[ ]	[x]	A full description of the statistical parameters including central tendency (e.g. means) or other basic estimates (e.g. regression coefficient) AND variation (e.g. standard deviation) or associated estimates of uncertainty (e.g. confidence intervals)
[ ]	[x]	For null hypothesis testing, the test statistic (e.g. *F*, *t*, *r*) with confidence intervals, effect sizes, degrees of freedom and *P* value noted<br>*Give P values as exact values whenever suitable.*
[x]	[ ]	For Bayesian analysis, information on the choice of priors and Markov chain Monte Carlo settings
[x]	[ ]	For hierarchical and complex designs, identification of the appropriate level for tests and full reporting of outcomes
[ ]	[x]	Estimates of effect sizes (e.g. Cohen's *d*, Pearson's *r*), indicating how they were calculated

Our web collection on statistics for biologists contains articles on many of the points above.

Software and code

Policy information about availability of computer code

Data collection

SRA Toolkit v3.0.0 was used to retrieve FASTQ files corresponding to samples other than those sequenced in this manuscript.

Data analysis

- fastp v0.20.0 was used for trimming and quality filtering of sequencing reads.
- BWA-MEM v0.7.4 was used to align sequencing reads to the reference genomes.
- Picard v2.6.0 was used to mark duplicate reads.
- GATK v4.1.1.0 was used for variant calling and to retrieve allele balance information.
- VCFtools v0.1.14 was used to calculate population genetic parameters.
- PLINK v1.9 was used to calculate identity-by-state (IBS), perform multidimensional scaling, and filter variants in linkage disequilibrium for use in ADMIXTURE.
- ADMIXTURE v1.3.0 was used to estimate the value of K with the lowest cross-validation error and to infer population structure.
- vcf2phylip v2.3 was used to generate haplotype sequences from VCF files.
- IQ-TREE v2.3.6 and RAxML v8.2.12 were used to construct phylogenies, and the R package ape v5.0 was used to analyze tree topologies.
- Microreact v.282 was used for phylogeny visualization.
- The R package vegan (2.7-2) was used to perform Mantel tests.
- RAxML v8.2.1252 was also used t reconstruct phylogenies.
- BCFtools 1.9 was used to estimate per variant heterozygosity.
- ape 5.0 was used to select true introgressions as those blocks that grouped with S. paradoxus in the phylogeny.
- GO Term Finder tool from the Saccharomyces Genome Database was used to perform Gene Ontology enrichment analysis.
- Visualization of ADMIXTURE results was done in Pong 1.4.9.
- ggplot 4.0.0

- rnaturalearth 1.1.0
- Custom Scripts: scripts for identifying introgressed genes, determining their origins and generating the figures presented in this study are available at:
Zenodo https://doi.org/10.5281/zenodo.17970285 (2025)

For manuscripts utilizing custom algorithms or software that are central to the research but not yet described in published literature, software must be made available to editors and reviewers. We strongly encourage code deposition in a community repository (e.g. GitHub). See the Nature Portfolio guidelines for submitting code & software for further information.

Data

Policy information about availability of data

All manuscripts must include a data availability statement. This statement should provide the following information, where applicable:

- Accession codes, unique identifiers, or web links for publicly available datasets
- A description of any restrictions on data availability
- For clinical datasets or third party data, please ensure that the statement adheres to our policy

Genome sequencing data generated in this study have been deposited in the NCBI SRA under the BioProject accession PRJNA1138754 [https://www.ncbi.nlm.nih.gov/bioproject/?term=PRJNA1138754]. The accession numbers of each genome employed in this study, including those previously sequenced, are provided in Supplementary Data 1 and Supplementary Data 2, together with their corresponding hyperlink. A Spanish translation of the article is provided in Supplementary Data 5. Source data are provided with this paper.

Research involving human participants, their data, or biological material

Policy information about studies with human participants or human data. See also policy information about sex, gender (identity/presentation), and sexual orientation and race, ethnicity and racism.

Reporting on sex and gender	NA
Reporting on race, ethnicity, or other socially relevant groupings	NA
Population characteristics	NA
Recruitment	NA
Ethics oversight	NA

Note that full information on the approval of the study protocol must also be provided in the manuscript.

Field-specific reporting

Please select the one below that is the best fit for your research. If you are not sure, read the appropriate sections before making your selection.

☐ Life sciences ☐ Behavioural & social sciences ☒ Ecological, evolutionary & environmental sciences

For a reference copy of the document with all sections, see nature.com/documents/nr-reporting-summary-flat.pdf

Ecological, evolutionary & environmental sciences study design

All studies must disclose on these points even when the disclosure is negative.

Study description	This study reports the genome sequences of 216 Saccharomyces cerevisiae isolates obtained from spontaneous open fermentations of agave must. We characterize their population genomics within the context of the known diversity of the species and provide a detailed assessment of introgression profiles across these newly sequenced strains, all of which belong to a single higher-level phylogenetic clade.
Research sample	We investigated the genetic diversity of Saccharomyces cerevisiae associated with spontaneous agave fermentations in Mexico. Strains were collected across multiple independent sampling efforts previously reported in the literature. To ensure broad geographic representation, we included isolates from all distilleries sampled across the seven agave spirit–producing regions. Most isolates were obtained from Gallegos-Casillas et al. (Yeast, 2024), with additional strains from Lachance (1995), Kirchmayr and Gschaedler (CIATEJ), and Maritza Alvarez (CIAD). Sample origins are detailed in Supplementary Figure 1 and Supplementary Table 1.
Sampling strategy	We did not calculate sample size, instead we sequenced at least one isolate from each distillery from Gallegos-Casillas et al. Yeast 2024 and the strains provided by collaborators from previous sampling efforts. We ensured that all major producing regions in Mexico were included.
Data collection	Strains and associated metadata were provided by the original collectors cited above. Their sequencing was done after purifying DNA with the MasterPure DNA 425 purification kit as recommended by the manufacturer, and was sequenced using DNBSeq 426 (BGI,

	China).
Timing and spatial scale	Most S. cerevisiae isolates were collected between 2018 and 2021 by Gallegos-Casillas et al. (Yeast, 2024), covering all relevant agave spirit–producing regions. Additional strains from earlier sampling efforts were also included to capture historical diversity: from 1988 (Kirchmayr & Gschaedler) and 1992 (Lachance) from the West-II region, 2013 from the northwest (Alvarez-Ainza), and other isolates obtained by CIATEJ between 2008 and 2016 from the Central Highlands, the SouthCentral region the Balsas Basin and again from West-II. Full sampling details are provided in Supplementary Figure 1 and Supplementary Table 1.
Data exclusions	No data were excluded from the analyses. One strain originally considered for inclusion was removed prior to sequencing at the request of collaborators due to intended commercial use.
Reproducibility	Phylogenomic classifications of the isolates were consistent across all analytical stages, with stable grouping patterns. While the precise relationships among some clades varied depending on the phylogenetic reconstruction strategy (as shown in Supplementary Figure S2). These differences did not affect overall conclusions. Introgression analyses produced consistent patterns across alternative definitions of introgressed genes and potential donor clades.
Randomization	No randomization was applicable. Geographic origin was assigned according to officially recognized agave spirit–producing regions, ensuring full regional representation. Further groupings were derived from phylogenomic and population structure analyses.
Blinding	Blinding was not applicable to this study, as analyses were based on genomic data and metadata without subjective interpretation.

Did the study involve field work? ☐ Yes ☒ No

Reporting for specific materials, systems and methods

We require information from authors about some types of materials, experimental systems and methods used in many studies. Here, indicate whether each material, system or method listed is relevant to your study. If you are not sure if a list item applies to your research, read the appropriate section before selecting a response.

Materials & experimental systems

n/a	Involved in the study	
☒	☐	Antibodies
☒	☐	Eukaryotic cell lines
☒	☐	Palaeontology and archaeology
☐	☒	Animals and other organisms
☒	☐	Clinical data
☒	☐	Dual use research of concern
☒	☐	Plants

Methods

n/a	Involved in the study	
☒	☐	ChIP-seq
☒	☐	Flow cytometry
☒	☐	MRI-based neuroimaging

Animals and other research organisms

Policy information about studies involving animals; ARRIVE guidelines recommended for reporting animal research, and Sex and Gender in Research

Laboratory animals	*For laboratory animals, report species, strain and age OR state that the study did not involve laboratory animals.*
Wild animals	*Provide details on animals observed in or captured in the field; report species and age where possible. Describe how animals were caught and transported and what happened to captive animals after the study (if killed, explain why and describe method; if released, say where and when) OR state that the study did not involve wild animals.*
Reporting on sex	*Indicate if findings apply to only one sex; describe whether sex was considered in study design, methods used for assigning sex. Provide data disaggregated for sex where this information has been collected in the source data as appropriate; provide overall numbers in this Reporting Summary. Please state if this information has not been collected. Report sex-based analyses where performed, justify reasons for lack of sex-based analysis.*
Field-collected samples	*For laboratory work with field-collected samples, describe all relevant parameters such as housing, maintenance, temperature, photoperiod and end-of-experiment protocol OR state that the study did not involve samples collected from the field.*
Ethics oversight	*Identify the organization(s) that approved or provided guidance on the study protocol, OR state that no ethical approval or guidance was required and explain why not.*

Note that full information on the approval of the study protocol must also be provided in the manuscript.

Plants

Seed stocks	*Report on the source of all seed stocks or other plant material used. If applicable, state the seed stock centre and catalogue number. If plant specimens were collected from the field, describe the collection location, date and sampling procedures.*
Novel plant genotypes	*Describe the methods by which all novel plant genotypes were produced. This includes those generated by transgenic approaches, gene editing, chemical/radiation-based mutagenesis and hybridization. For transgenic lines, describe the transformation method, the number of independent lines analyzed and the generation upon which experiments were performed. For gene-edited lines, describe the editor used, the endogenous sequence targeted for editing, the targeting guide RNA sequence (if applicable) and how the editor was applied.*
Authentication	*Describe any authentication procedures for each seed stock used or novel genotype generated. Describe any experiments used to assess the effect of a mutation and, where applicable, how potential secondary effects (e.g. second site T-DNA insertions, mosiacism, off-target gene editing) were examined.*
