# Supplementary material for: Recurrent introgression and geographical stratification shape Saccharomyces cerevisiae in the Neotropics
Source: Nat Commun. 2026 Feb 21;17:3024. doi: 10.1038/s41467-026-69138-0 (PMC13035892; doi:10.1038/s41467-026-69138-0)
Supplement: Supplementary file 4 — Description of Additional Supplementary Files [file 41467_2026_69138_MOESM4_ESM.pdf]

Description of Additional Supplementary Files

Supplementary Data 1

Description: List of the 216 strains sequenced in this study, with associated sampling and genomic metadata

Supplementary Data 2

Description: List of strains and associated metadata used in this study. This dataset includes the 216 strains sequenced here; 1,010 strains from Peter et al. (2018); SAM group strains from Tellini et al. (2024), comprising 23 strains from Barbosa et al. (2016), 10 from Barbosa et al. (2018), 3 from Gallone et al. (2016), and 1 from Legras et al. (2018); and 21 strains from the Alpechin clade described by Pontes et al. (2019).

Supplementary Data 3

Description: Statistical tests of overlap enrichment among introgressed gene sets from Neotropical clades. Pairwise group comparisons were performed using one-sided Fisher's exact tests to assess enrichment beyond expectation. Overlaps involving more than two groups were evaluated using one-sided upper-tail cumulative hypergeometric tests.

Supplementary Data 4

Description: List of introgressed genes identified in this study, with associated metadata.

Supplementary Data 5

Description: Spanish translation of the article
