# Supplementary material for: Recurrent introgression and geographical stratification shape Saccharomyces cerevisiae in the Neotropics
Source: Nat Commun. 2026 Feb 21;17:3024. doi: 10.1038/s41467-026-69138-0 (PMC13035892; doi:10.1038/s41467-026-69138-0)
Supplement: Supplementary file 9 — Supplementary Data 5 [file 41467_2026_69138_MOESM9_ESM.pdf]

VERSIÓN EN ESPAÑOL

Esta versión en español fue traducida por los autores a partir de la versión original en inglés usando la herramienta de traducción de documento completo de Word Office para Mac Versión 16.103.2, y posteriormente haciendo una edición manual para corregir errores gramaticales, mejorar la sintaxis e interpretación del texto original.

Introgresiones recurrentes y estratificación geográfica determinan a *Saccharomyces cerevisiae* en el Neotrópico

J. Abraham Avelar-Rivas[1^], Iván Sedeño[2,3], Luis F. García-Ortega[4], Jose A. Urban Aragon[2,3‡], Claudio López-Gallegos[1,4], Xitlali Aguirre-Dugua[5], Eugenio Mancera[4,#,*], Alexander DeLuna[1,6,#,*], Lucia Morales[2,#,*]

[1] *Unidad de Genómica Avanzada, Centro de Investigación y de Estudios Avanzados del Instituto Politécnico Nacional, Irapuato, México*

[2] *Laboratorio Internacional de Investigación sobre el Genoma Humano (LIIGH), Universidad Nacional Autónoma de México, Querétaro, México*

[3] *Posgrado en Ciencias Biológicas, Universidad Nacional Autónoma de México, Coyoacán, CDMX, México*

[4] *Departamento de Ingeniería Genética, Unidad Irapuato, Centro de Investigación y de Estudios Avanzados del Instituto Politécnico Nacional, Irapuato, México*

[5] *Investigadoras e Investigadores por México, Secretaría de Ciencia, Humanidades, Tecnología e Innovación, México, México*

[6] *Centro de Investigación sobre el Envejecimiento, Centro de Investigación y de Estudios Avanzados del Instituto Politécnico Nacional, Tlalpan, CDMX, México*

^Adscripción actual: CNRS, INSERM, IRCAN, Côte d'Azur University, Nice, France

‡ Adscripción actual: Department of Human Genetics, University of Chicago, Chicago, IL, USA

[#]*Estos autores supervisaron conjuntamente este trabajo:* Eugenio Mancera, Alexander DeLuna y Lucía Morales

[*]*Correspondencia con:* Eugenio Mancera (eugenio.mancera@cinvestav.mx), Alexander DeLuna (alexander.deluna@cinvestav.mx) y Lucia Morales (lmorales@liigh.unam.mx)

RESUMEN

Desde las levaduras hasta los humanos, la hibridación introgresiva influye significativamente en la historia evolutiva de los organismos vivos al introducir nueva diversidad genética. Las cepas de *Saccharomyces cerevisiae* en todo el mundo muestran introgresiones de su especie hermana *S. paradoxus*, a pesar de que la identidad promedio entre los genomas de estas especies es inferior al 90%. Aunque los aislados de *S. cerevisiae* del Neotrópico son conocidos por sus altos niveles de introgresión, los eventos de hibridación que los originan siguen siendo desconocidos. En este estudio secuenciamos 216 cepas de *S. cerevisiae* de fermentaciones abiertas y espontáneas de agave en todo México. Los genomas de estas cepas revelan una considerable diversidad genética y una estructura poblacional vinculada a la distribución geográfica que se había pasado por alto debido a que esta región megadiversa estaba submuestreada. Estas cepas, junto con las de la Guayana Francesa, Ecuador y Brasil, forman un grupo filogenético Neotropical más amplio que está notablemente enriquecido en introgresiones. Sorprendentemente, los orígenes de las introgresiones y sus patrones de conservación indican múltiples eventos de hibridación, lo que sugiere barreras flexibles entre especies en esta región. Nuestros hallazgos subrayan procesos evolutivos simultáneos—estratificación geográfica y múltiples introgresiones—que moldean los genomas de este linaje diverso de *S. cerevisiae*. Las levaduras neotropicales representan un laboratorio natural para explorar los mecanismos y la importancia adaptativa de la hibridación introgresiva en la evolución del genoma eucariota.

INTRODUCCIÓN

Más allá de su estatus consolidado como el modelo de células eucariotas más estudiado en el laboratorio, la levadura común *Saccharomyces cerevisiae* se ha convertido en un modelo sobresaliente para estudios en ecología, genómica de poblaciones y evolución[1–5]. Los análisis genómicos de miles de aislados de entornos naturales y antropogénicos en todo el mundo revelan que la especie está estructurada en clados que se correlacionan ampliamente con la geografía y el sustrato de donde fueron aislados[6–8]. Estos estudios genómicos globales también han demostrado que la mayoría de las cepas albergan material genético de otras especies de *Saccharomyces*, fenómeno conocido como introgresión genética. Por ejemplo, la mayoría de las cepas de *S. cerevisiae* de todo el mundo albergan genes introgresados de la especie hermana *Saccharomyces paradoxus*[9,10,6,11,12], aunque estas especies divergieron hace 4-6 millones de años y tienen una divergencia nucleotídica media de ~14%[13,14].

La región Neotropical, que se extiende desde el norte de México hasta Sudamérica, es sumamente relevante desde el punto de vista biogeográfico debido a sus altos índices de biodiversidad[15]. Algunas de las cepas de *S. cerevisiae* de esta área se agrupan en una larga rama monofilética previamente considerada como compleja por Pontes *et al.* (2020)[7]. Esta rama agrupa cuatro clados: Mexican Agave (MA), French Guiana (FG), Wild Brazil 3 (WB3) y South American Mix 2 (SAM2). Las cepas WB3 se aislaron de áreas naturales, mientras que la mayoría de las cepas de este grupo Neotropical fueron aisladas de ambientes antropogénicos. Teniendo en cuenta la vasta extensión que abarca esta región, los pocos aislados secuenciados disponibles probablemente subestiman la diversidad genética, limitando así nuestro conocimiento de la especie en esta parte del mundo.

El puñado de genomas secuenciados disponibles indica que el grupo Neotropical de *S. cerevisiae* alberga un número inusualmente alto de introgresiones de *S. paradoxus*[16,6,7,17,12]. Esto podría explicar la alta divergencia de este grupo en comparación con otros clados. Una fracción de las introgresiones del grupo Neotropical se ha asociado con poblaciones de *S. paradoxus* de América[6,12,16], lo

que contrasta con el origen de las introgresiones presentes en linajes europeos[6,18]. Incluso se ha sugerido que las introgresiones en las cepas brasileñas derivan de dos diferentes poblaciones de *S. paradoxus*, lo que aumenta la posibilidad de múltiples eventos de hibridación[16]. Sin embargo, los orígenes precisos de las introgresiones estaban poco claros, en parte debido al número limitado de secuencias genómicas de *S. cerevisiae* y *S. paradoxus* disponibles para esta región. En México, se ha reportado la presencia de *S. paradoxus* con baja frecuencia en fermentaciones de agave[19–23], incluyendo un linaje que coexiste con *S. cerevisiae* en los alrededores de las destilerías de agave, que no ha sido considerado en estudios previos[22]. Este linaje podría estar posiblemente asociado a las introgresiones en la América tropical. Un entendimiento integral de la evolución de *S. cerevisiae* y el origen de sus introgresiones es esencial para determinar con precisión la dinámica del flujo génico interespecífico en la región.

Para explorar la diversidad genómica de *S. cerevisiae* en América tropical—especialmente a la luz de sus fuertes huellas de introgresión—secuenciamos 216 cepas aisladas principalmente de fermentaciones tradicionales de agave en México. Estas fermentaciones espontáneas son sistemas antropogénicos y abiertos que dependen de microorganismos de los entornos circundantes, a menudo naturales[22,23]. Estudios previos han puesto de manifiesto la megadiversidad en esta región[24] así como una alta diversidad de *S. cerevisiae* en la fermentación de agave[25–28], pero los esfuerzos de secuenciación de genomas completos sólo han incluido nueve cepas hasta la fecha, la mayoría de una sola región productora de destilados de agave[6]. En este trabajo, mostramos que la mayoría de los aislados que secuenciamos pertenecen al grupo Neotropical, revelando nuevos clados donde la diversidad se correlaciona con el origen geográfico de las cepas. Además, al analizar los patrones y orígenes de las introgresiones en el contexto de la relación entre estos linajes, identificamos múltiples eventos de introgresión en su historia evolutiva. Notablemente, algunos de estos pulsos de introgresión son exclusivos de cepas aisladas de fermentaciones de agave. Nuestra refinada filogenia nos permite ilustrar cómo

ocurren episodios recurrentes de flujo genético entre dos especies hermanas del género *Saccharomyces* en una región megadiversa del mundo.

RESULTADOS

Para ofrecer una visión completa de la diversidad de *S. cerevisiae* en una de las regiones más biodiversas del mundo[15,24], secuenciamos los genomas de 216 aislados provenientes mayormente de destilerías artesanales de agave a lo largo de México (**Figura S1**, **Datos Suplementarios S1**). Este entorno de fermentación antropogénica representa un buen punto de partida para estudiar la diversidad de levaduras, ya que las fermentaciones espontáneas se han producido durante más de 3,500 años en una amplia variedad de ecosistemas, latitudes y contextos culturales[29–31]. En concreto, 211 de las cepas secuenciadas fueron aisladas de fermentaciones de agave mediante diversos esfuerzos de muestreo realizados en todo el país entre los años 1988 y 2021[32,25,33,28,34,26,22,20]. Cuatro de las cinco cepas restantes fueron obtenidas de fermentaciones tradicionales de otros sustratos —pulque (fermentación de savia de agave cruda), sidra de manzana, tunas y tallos cocidos de plantas de sotol (*Dasylirion* spp.)— mientras que la quinta era una cepa de levadura comercial no caracterizada y utilizada en una de las destilerías muestreadas; todas ellas fueron aisladas en México (**Datos Suplementarios S1**). Con los 216 genomas (cobertura promedio de profundidad = 163X), evaluamos la ubicación filogenética y la estructura poblacional de las cepas en el contexto de la diversidad conocida de la especie, así como la dinámica y el origen de las introgresiones preponderantes de *S. paradoxus* dentro de cada población.

Las cepas de fermentación de agave pertenecen a un grupo Neotropical genéticamente diverso

Para evaluar el parentesco entre las cepas *S. cerevisiae* provenientes de fermentaciones de agave y otros aislados de la especie, primero realizamos un análisis de escalamiento multidimensional (MDS por sus siglas en inglés) con 1,262 genomas, incluyendo las 216 cepas recién secuenciadas. La mayoría de los aislados de México se agruparon con otras cepas de América Tropical, como las de

los clados French Guiana (FG), Wild Brazil 3 (WB3), South American Mix 2 (SAM2) y Mexican Agave 1 (MA1, anteriormente llamada “Mexican Agave”) (**Figura 1a).** La mayoría de las cepas aisladas en el trópico del continente americano forman un grupo consolidado, separado de otros clados del mundo (**Figura 1a**), incluso cuando las cepas de fermentación de agave no están sobrerrepresentadas en el análisis MDS (**Figura S2**). Sólo once de las cepas secuenciadas en este estudio se agruparon en clados fuera del grupo Neotropical, mientras que los cuatro aislados que secuenciamos de otros sustratos se agruparon con cepas de fermentación de agave.

Para establecer las relaciones filogenéticas de los aislados que secuenciamos, los comparamos con un panel de cepas de referencia (véase **Datos Suplementarios S2**). De forma consistente con el análisis MDS, la filogenia mostró que 205 de las 216 cepas secuenciadas en este estudio (94,9%) forman parte de un grupo monofilético que incluye dos nuevos clados, Mexican Agave 2 (MA2) y Tequila Distillery, junto con los clados previamente definidos MA1, FG, WB3 y SAM2 (**Figura S2**). La diferenciación genética de MA1, MA2, WB3 y FG fue validada usando un análisis de *Fst* ponderado (**Figura S2**). Decidimos llamar a este nuevo grupo filogenético el grupo Neotropical de *S. cerevisiae*, dado que casi todas las cepas de este grupo provienen de América tropical (**Figura 1b**). Dentro de las cepas secuenciadas que no se agruparon dentro del grupo Neotropical , cinco se agruparon con el clado North American Oak, que comúnmente se considera silvestre[6,8,35]. Otros cinco aislados —incluida la cepa comercial— están estrechamente relacionados con el clado Mixed Origin y solo uno se agrupa con las cepas del clado Wine.

El grupo Neotropical mostró consistentemente una rama relativamente larga en la filogenia, abarcando todas las cepas de seis clados: MA1, MA2, FG, WB3, Tequila Distillery y SAM2. La rama larga se vio respaldada por el elevado número de variantes de un solo nucleótido (SNV) presentes en las cepas de estos clados (**Figura 2**). En algunas de las reconstrucciones filogenéticas, la inclusión de las cepas mosaico de Tequila Distillery y SAM2 cambió la ubicación relativa de MA1,

MA2, WB3 y FG (**Figura S2**). Estas y otras cepas mosaico fueron excluidas de la filogenia principal (**Figura 1b**) para evitar desplazamientos topológicos artificiales producidos por la mezcla. Para determinar si las longitudes de las ramas observadas en el grupo Neotropical estaban determinadas por regiones introgresadas, generamos nuevamente la filogenia excluyendo las introgresiones (Métodos). La eliminación de estas regiones no tuvo impacto en la topología general de la filogenia y sólo mostró un efecto modesto en la longitud de las ramas del grupo Neotropical (**Figura S2**). El número de SNVs de los clados neotropicales disminuyó ligeramente cuando se eliminaron las regiones introgresadas (**Figura 2**). En contraste, la eliminación de la introgresión redujo notablemente la longitud de las ramas y el número de SNV en el clado de Alpechin, un grupo de cepas europeas que alberga un alto número de introgresiones de *S. paradoxus*. Para evaluar si las ramas largas eran atribuibles al sesgo en la cantidad de cepas muestreadas, reconstruimos las filogenias con base en un muestreo aleatorio considerando un máximo de cinco cepas por clado (**Figura S2**). Las topologías filogenéticas resultantes mostraron consistentemente la misma posición y ramas relativamente largas del grupo Neotropical, aunque el conjunto de datos más equilibrado redujo ligeramente la longitud relativa de las ramas (**Figura S2**), lo cual coincide con un informe anterior[35]. En conjunto, estos resultados destacan la robustez de los clados dentro del grupo Neotropical y su característica rama larga, consistente con las filogenias globales previamente reportadas de *S. cerevisiae*[6,8].

Como se mencionó anteriormente, pudimos distinguir tres clados entre los aislados recién secuenciados de fermentaciones de agave en el grupo Neotropical de *S. cerevisiae*. Primero, identificamos un grupo de 20 aislados del noreste de México, que incluía las siete cepas previamente reportadas como “Mexican Agave”[6], en adelante denominado clado “Mexican Agave 1” (MA1) (**Figura 1b**). Cabe mencionar que el clado monofilético MA1 comprende hasta ahora sólo cepas aisladas al noreste de la Sierra Madre Oriental, una cordillera que discurre paralela a la costa del Golfo de México y actúa como barrera natural, ejerciendo una fuerte influencia sobre el clima y la biodiversidad a ambos lados de la cordillera[36,37]. Como resultado del muestreo exhaustivo de los aislados aquí reportados, identificamos un nuevo

clado MA2 estrechamente relacionado con MA1 (**Figura 1b**). La mayoría de las cepas recién secuenciadas en este estudio (81,9%) pertenecen al clado MA2. Dos cepas previamente secuenciadas aisladas en México[6] que habían sido asignadas al clado SAM2[12], fueron reclasificadas tras nuestros análisis dentro del nuevo clado MA2. El tercer grupo de aislados de agave está compuesto por 15 cepas, la mayoría con genotipos mezclados, todas secuenciadas en este estudio (**Figura 1b**, **Figura S2**). Nos referimos a este grupo como “Tequila Distillery”, ya que once de las 15 cepas fueron aisladas de la industria del tequila.

Nuestros resultados muestran que la mayoría de las cepas de fermentaciones espontáneas de agave forman parte del grupo Neotropical, revelando que el grupo previamente descrito como “Mexican Agave” representa solamente una fracción de la diversidad genética presente en las fermentaciones de agave. Además, los clados dentro de este grupo, incluidos los grupos recién identificados, se sitúan entre los más altos en términos de SNVs en comparación con el genoma de referencia, superados únicamente por los clados asiáticos silvestres (**Figura 2**, **Figura S3**). Esta tendencia se mantuvo cuando se cuantificó la identidad una a una para cada secuencia (**Figura S3**), lo que indica que es independiente del genoma de referencia específico utilizado. La alta divergencia genética de las cepas neotropicales fue recientemente respaldada por una comparación por pares de 3,334 genomas de *S. cerevisiae* que revelaron que la mayor distancia genética por pares dentro de la especie se encuentra entre un aislado taiwanés silvestre y una cepa mexicana de agave[8]. En conjunto, nuestros análisis filogenómicos y poblacionales de *S. cerevisiae* de América tropical cierran una brecha en el conocimiento genético de las especies en esta región.

Las poblaciones de levaduras neotropicales están estratificadas por geografía

Las levaduras de América tropical provienen de una gran variedad de ambientes a lo largo de una amplia gama de distancias geográficas. Por ello, nos preguntamos hasta qué punto los parámetros geográficos influyen en la estructura poblacional de

S. cerevisiae en el grupo Neotropical. Para ello realizamos un análisis ADMIXTURE, seleccionando el número de poblaciones que mejor explicaban los genotipos en función del menor error de validación cruzada (**Figura 3a**, **Figura S4**). El análisis de la estructura poblacional reveló once componentes ancestrales dentro del grupo Neotropical. Específicamente, las cepas de los clados MA1, Tequila Distillery, FG y WB3 formaron poblaciones distintas, mientras que las siete poblaciones restantes procedían del clado MA2 recién identificado.

Dada la estratificación observada en el grupo Neotropical, analizamos la distribución geográfica de estos aislados según su estructura poblacional. Los once colores en el gráfico de ADMIXTURE que representan componentes ancestrales dentro del grupo Neotropical mostraron buena concordancia con su distribución geográfica (**Figura 3a**). Esta estratificación geográfica se observó en dos niveles de resolución. Primero, cada uno de los cinco clados del grupo Neotropical está restringido a regiones específicas en el mapa. Segundo, dentro de la resolución del clado MA2, los siete colores del ADMIXTURE también se encuentran estratificados geográficamente. En general, las cepas de la misma región productora de destilados de agave se agrupan juntas en la filogenia y tienen el mismo componente poblacional ancestral. Esto también se demostró mediante una correlación entre las distancias geográficas y genéticas en este conjunto de cepas (r=0.18, significancia=0.0001 con 9,999 permutaciones; prueba de Mantel de Spearman, unilateral, incluyendo las cepas MA1 y MA2). También observamos un aumento de la diversidad genética y la heterocigosidad de norte a sur dentro del clado MA2 (**Figura S5**). En conjunto, estos hallazgos sugieren que la geografía influye en la diversidad genómica en el grupo Neotropical.

A pesar de la relación general entre la geografía y la estructura poblacional en las cepas de América tropical, hubo excepciones que sugieren flujo génico dentro e incluso más allá del linaje neotropical. Por ejemplo, una cepa aislada de una fermentación espontánea de agave compartía ascendencia cercana con aislados silvestres del clado North American Oak, mientras que otros cuatro aislados estrechamente relacionados con el clado North American Oak mostraron signos de

mezcla con diferentes componentes genéticos del grupo Neotropical (**Figura 3a**). El clado WB3 mostró poca mezcla, mientras que las cepas de SAM2 tenían componentes genéticos de poblaciones de MA2, FG y WB3, lo cual no es sorprendente ya que son parientes cercanos. Además, diez cepas del clado Tequila Distillery mostraron mezcla, principalmente entre diferentes poblaciones neotropicales, pero también con otros clados como el de vino (Wine). En conjunto, estos hallazgos sugieren que las poblaciones de *S. cerevisiae* en América tropical se han separado, al menos parcialmente, por aislamiento geográfico, mientras que otros procesos ecológicos o factores relacionados con el ser humano han promovido el flujo génico, moldeando también la estructura genética de la población.

Utilizamos los datos genómicos para explorar más a fondo los patrones de diversidad genética en las cepas neotropicales. Cada clado difiere significativamente en diversidad nucleotídica (π), *D* de Tajima y heterocigosidad (**Figura 3b-d**, **Figura S6**), algunos mostrando alta diversidad genética a pesar de que la mayoría de sus cepas están aisladas de ambientes antropogénicos. Por ejemplo, los clados MA1, MA2 y Tequila Distillery presentan mayor diversidad nucleotídica que el clado Wine e incluso que el altamente introgresado clado Alpechin (**Figura S6**). Estos hallazgos sugieren que la elevada diversidad genética observada en los clados relacionados con agave no puede atribuirse únicamente a las introgresiones. En contraste, el clado FG mostró baja diversidad genética y un grado homogéneo de heterocigosidad. El clado Tequila Distillery mostró los niveles más altos de heterocigosidad y diversidad de nucleótidos, en parte debido a los genotipos mezclados de sus cepas, y en consonancia con las tendencias observadas en aislados de cerveza[6]. En conjunto, nuestros análisis de estructura poblacional y diversidad genética indican que las levaduras de América tropical constituyen poblaciones diversas y estructuradas, estratificadas por la geografía.

Las diferencias en la distribución de las introgresiones revelan un flujo génico recurrente entre especies

Los genomas de las cepas de *S. cerevisiae* dentro del grupo Neotropical se caracterizan por un número atípicamente alto de regiones introgresadas de *S.*

paradoxus[12,16]. Para obtener una visión sobre la dinámica del flujo génico, identificamos el conjunto de genes introgresados con una estrategia que utiliza el mapeo competitivo de lecturas de secuenciación a ambos genomas parentales y análisis filogenéticos (Métodos). Es importante destacar que la mayoría de las introgresiones que identificamos también se encontraron utilizando otras estrategias, incluyendo: i) ensamblaje genómico seguido de un enfoque filogenético[6], ii) identificando marcadores diagnósticos *bona fide* de introgresiones de un solo nucleótido[12] y iii) filtrando ortogrupos después de realizar el mapeo competitivo[16] (**Figura S7**).

Todos los clados neotropicales mostraron más genes introgresados que los demás grupos del mundo, excepto el clado Alpechin (**Figura 4a**). Los tres clados de Sudamérica (WB3, SAM2 y FG) mostraron introgresiones en menor número y más cortas, con una fracción menor de genes heterocigotos introgresados, en comparación con los tres clados de agave (Tequila Distillery, MA1 y MA2) (**Figura 4a**, **Figura S8**). Aquí, llamamos heterocigoto a un gen introgresado cuando tanto el alelo de *S. cerevisiae* como el alelo de *S. paradoxus* están presentes dentro del mismo genoma (**Figura 4a**, **Figura S8**, **Figura S9**).

Entre los clados neotropicales, encontramos que los aislados de MA1 mostraron tanto el mayor número como el rango más amplio en el número de genes introgresados, con recuentos que van desde 94 hasta 320 genes por cepa. Los aislados MA2 presentaron entre 64 y 122 genes introgresados (**Figura 4A**). Además, identificamos un subconjunto de cepas del clado MA1 con más de 150 genes introgresados que presentan introgresiones más largas y heterocigotas que la mayoría de las cepas del clado (**Figura 4**, **Figura S8**).

Para inferir la historia de los genes introgresados, analizamos sus patrones de retención dentro del mismo clado y con respecto a otros. Observamos que los patrones de presencia–ausencia agrupan a los tres clados asociados al agave y los separan claramente de un segundo clúster que incluye a los tres grupos sudamericanos (**Figura 4b**, **Datos Suplementarios S3, Figura S9**). A pesar de las diferencias entre estos tres grupos, identificamos un sobrelape significativo en la

mayoría de las comparaciones entre subconjuntos de los clados neotropicales (**Datos Suplementarios S3**), incluidos cinco genes que son comunes a los seis clados neotropicales. Este número de genes compartidos introgresados supera el número esperado por azar (prueba hipergeométrica, unilateral, p = 0,00234; **Datos Suplementarios S3**). Esto probablemente se explique por un pulso de introgresión en el ancestro común de todas las cepas neotropicales, aunque una fuerte selección también podría explicar la presencia de este conjunto compartido de genes introgresados. Sin embargo, no se detectó un enriquecimiento significativo de GO entre los genes presentes en al menos el 75% de las cepas de cada clado. Como se esperaba, el análisis de los patrones de presencia-ausencia de genes introgresados en el clado de Alpechin, utilizado como referencia, no reveló un sobrelape significativo con los clados neotropicales, lo que confirma que en este grupo ocurrió un evento de hibridación independiente como se había reportado previamente [12,18].

Dados los complejos patrones de introgresión observados en el clado MA1 —distribución multimodal del número de genes introgresados (**Figura 4a**), presencia de grandes segmentos heterocigotos introgresados (**Figura S8**) y número inusualmente alto de genes exclusivos de este clado pero presentes en baja frecuencia dentro de él (**Figura 4b**)— examinamos los patrones de retención junto con las relaciones filogenéticas de las cepas MA1 (**Figura S9**). Aunque la mayoría de las cepas compartía la mayoría de sus regiones introgresadas con aislados estrechamente relacionados filogenéticamente, siete cepas MA1 mostraron segmentos introgresados heterocigotos que se encontraron exclusivamente dentro de grupos filogenéticos restringidos. Este patrón podría explicarse por múltiples pérdidas convergentes de los mismos genes introgresados en otras ramas de MA1 y en MA2, o más probablemente, por al menos un evento adicional de flujo génico interespecífico de *S. paradoxus* que ocurrió exclusivamente en MA1.

Las introgresiones en el grupo Neotropical provienen de diferentes linajes de *S. paradoxus*

Para rastrear los orígenes de las regiones introgresadas observadas, inferimos cuál era el linaje de *S. paradoxus* más probablemente asociado a cada gen. Para ello, evaluamos la identidad de secuencia de cada bloque introgresado contra un panel de genomas de *S. paradoxus* representativos de América. Este panel incluye un conjunto diverso de cepas que representan a las poblaciones previamente definidas de Norteamérica (*SpA*, *SpB SpC* y *SpD*), así como una cepa *SpB* de Brasil (*SpB_Bra*), junto con cepas representativas de otros clados de China, el Lejano Oriente Asiático, Hawái y la cepa de referencia del linaje europeo (aquí agrupada con *SpA*). Además, incluimos todas las cepas secuenciadas de México, abarcando tres linajes exclusivos de esta región: *SpB_MxAgave*, *SpB_Mx1* y *SpB_Mx2*[22]. Nuestros resultados indican que la mayoría de los genes introgresados en los clados mexicanos de agave de *S. cerevisiae* provienen de *SpB_MxAgave* (**Figura 5a**), una población que se encuentra exclusivamente en destilerías de agave en México. Una pequeña parte de los genes introgresados en estas cepas parece derivar de *SpB_Mx2* y del linaje *SpB* más amplio. En cambio, la mayoría de los genes introgresados en las cepas sudamericanas se rastrearon hasta el linaje *SpB* que se encuentra comúnmente en ambientes naturales en el Neotrópico, pero no se ha reportado en fermentaciones de agave[22]. Como era de esperar, los genes introgresados en el grupo Alpechin se parecen más a los del linaje europeo, previamente identificados como la población parental en este clado (**Table S4**)[38,18,12]. Estos hallazgos indican que al menos dos linajes de *S. paradoxus* se han hibridado con ancestros de las cepas neotropicales actuales, lo que resalta el papel de las interacciones interespecie en la configuración de la historia evolutiva de *S. cerevisiae* en la región.

DISCUSIÓN

La secuenciación exhaustiva de genomas *de S. cerevisiae* en regiones megadiversas y submuestreadas es esencial para comprender la diversidad genómica de esta especie, su historia evolutiva natural y su asociación con la domesticación. En este estudio, secuenciamos 216 cepas de fermentaciones abiertas de agave, generando un extenso conjunto de datos genómicos

poblacionales de aislados de ambientes antropogénicos en el Neotrópico. Nuestros análisis filogenéticos y de estructura poblacional revelan múltiples eventos de flujo génico interespecie que resultan en introgresiones dentro de este clado complejo que se encuentra geográficamente estructurado.

Las introgresiones afectan las trayectorias evolutivas a lo largo del árbol de la vida, desde bacterias hasta humanos [39,40], y son centrales en la historia evolutiva de *S. cerevisiae*[6,10–12]. En América, varios linajes de *S. cerevisiae* presentan un alto número de introgresiones de *S. paradoxus*, tanto en ambientes silvestres como antropogénicos[16,41,7,42,12]. Aquí ofrecemos una amplia visión de la dinámica evolutiva de la introgresión que ocurre en la región tropical de América con al menos tres pulsos de introgresiones. Probablemente se produjo una introgresión temprana en el ancestro común del grupo Neotropical antes de su diversificación, donde los genes de los linajes americanos *SpB* de *S. paradoxus* introgresaron en *S. cerevisiae* (**Figura 5b**). Este primer pulso fue seguido por un posterior evento de introgresión de otro linaje, *SpB_Mx*, en el antepasado común de MA1 y MA2. La presencia de grandes regiones heterocigotas introgresadas exclusivas de un subconjunto de cepas MA1 sugiere un pulso adicional y más reciente de introgresión exclusivo de este clado. Si estos pulsos múltiples de introgresión son resultados estocásticos de la coexistencia de *S. cerevisiae* y *S. paradoxus* en la región, o si reflejan procesos adaptativos, sigue sin estar claro, especialmente teniendo en cuenta que no encontramos enriquecimiento para categorías funcionales específicas entre los genes introgresados comúnmente conservados dentro de los clados.

El número e identidad de los genes introgresados varían mucho entre cepas MA1. Esto es coherente con una transición continua de hibridación a introgresión, probablemente moldeada por la inestabilidad genómica y las retrocruzas[18]. La variación en el contenido de genes introgresados puede deberse a la retención alélica diferencial durante la inestabilidad genómica, un diferente número de eventos de retrocruzas y el cruzamiento de diferentes cepas en distintas etapas de la transición de hibridación a genes introgresados estables. En general, los patrones variables de los genes introgresados en MA1 son difíciles de conciliar con la

retención diferencial del evento de hibridación compartido con MA2 o menos eventos de retrocruzamiento, aunque esto no puede descartarse del todo. Las pruebas experimentales y computacionales pueden ayudar a estimar el momento y la contribución relativa de estos escenarios, mientras que un muestreo más amplio en esta región probablemente también proporcionaría una visión más profunda de los procesos evolutivos que moldean el flujo génico interespecífico en *Saccharomyces*.

Nuestro esfuerzo de secuenciación amplió el conocimiento sobre la diversidad y biogeografía de *S. cerevisiae*. Identificamos dos clados adicionales dentro del grupo Neotropical: MA2 y Tequila Distillery. Junto con MA1, FG, WB3 y SAM2, el grupo Neotropical incluye en total seis clados distintos. Los análisis filogenéticos indican que los dos clados mexicanos de agave —MA1 y MA2— son clados hermanos, que a su vez son los parientes más cercanos de las cepas WB3 y FG, en concordancia con la filogenia previamente reportada[43]. La marcada heterogeneidad observada entre los pocos aislados de los grupos neotropicales submuestreados sugiere la existencia de linajes adicionales, aún no descubiertos, en América tropical, una región de diversidad y complejidad biogeográfica excepcionales[15]. Para comprender a plenitud la gran diversidad de *S. cerevisiae* en la región tropical de América y las subyacentes fuerzas evolutivas que determinan su historia, es esencial ampliar el muestreo ambiental y los análisis genómicos en ella.

Demostramos que los segmentos introgresados aumentan la longitud de las ramas en la filogenia, pero la longitud de las ramas del grupo Neotropical es menos afectada por la eliminación de introgresiones que la del grupo de Alpechin. Aunque la longitud de las ramas también puede verse influenciada por el total de cepas consideradas en la filogenia, el grupo Neotropical permaneció como uno de los linajes más divergentes al balancear el número de cepas por clado (**Figura S2**). La rama larga del grupo Neotropical puede deberse a múltiples factores, incluyendo una tasa elevada de mutación causada por un fenotipo de hipermutante[44] o

exposición a mutágenos, así como procesos demográficos como cambios en el tamaño efectivo de las poblaciones o un aumento de las tasas de exogamia.

Es importante destacar que la fracción de la diversidad genética previamente desconocida del grupo Neotropical está estructurada geográficamente. La influencia geográfica que moldeó la diversidad genética es evidente en varias dimensiones: primero, se observó una correlación clara entre cada uno de los once componentes ancestrales de las cepas del Neotrópico y su región de procedencia. En segundo lugar, las cepas del clado MA2 presentan una mayor diversidad nucleotídica y heterocigosidad, en un gradiente norte-sur. Finalmente, la divergencia genética entre los clados MA1 y MA2 (*Fst*=0,43, SII mediana=0,97) está asociada con la presencia de la cordillera Sierra Madre Oriental en México, que se encuentra en el borde donde coinciden las regiones biogeográficas Neotropical y Neártica. Se sabe que esta cadena montañosa actúa como una barrera geográfica que afecta la distribución de múltiples especies[15,36,37].

Durante las fermentaciones tradicionales de agave, la fermentación tiene lugar espontáneamente en tanques abiertos[23]. Las cepas de *S. cerevisiae* secuenciadas que provienen de fermentaciones de agave se han clasificado como domesticadas basándose en su fuente de aislamiento y en ciertas huellas genéticas[7,17]. Sin embargo, los límites entre las comunidades microbianas fermentadoras y las de los entornos circundantes suelen difuminarse en estos y otros sistemas abiertos. Por ejemplo, los aislados recolectados de *S. cerevisiae* de ambientes naturales adyacentes a destilerías son genéticamente similares a los de fermentaciones espontáneas abiertas de agave dentro de la misma zona[22]. De hecho, algunos aislados de las fermentaciones de agave se agrupan con cepas del clado North American Oak, típicamente clasificado como silvestre[6,8,35]. Además, ciertas subpoblaciones provenientes de fermentaciones de agave dentro del clado MA2 son predominantemente homocigotas, lo cual es un rasgo genético típicamente asociado a poblaciones silvestres. En conjunto, estos hallazgos sugieren que los límites entre cepas *S. cerevisiae* de las fermentaciones de agave y las de los hábitats naturales circundantes son difusos.

Más allá de los factores biogeográficos, las actividades humanas pueden haber desempeñado un papel en la configuración de las poblaciones de levaduras, como lo demuestran los eventos de cruzas dentro y fuera del grupo Neotropical. Queda por abordar hasta qué punto la estructura poblacional observada está impulsada por factores naturales como la ecología y biogeografía de los reservorios vegetales y vectores de insectos, o por dinámicas y prácticas humanas. Por ejemplo, prácticas específicas de producción a nivel local y la notable diversidad de sustratos de fermentación, con más de 50 diferentes especies de *Agave* utilizadas en la producción de destilados[45], pueden promover la diferenciación poblacional. Anteriormente se ha demostrado que la composición de las comunidades microbianas en las fermentaciones de agave está determinada por factores locales [46]. Con la creciente demanda mundial de destilados de agave, existe un riesgo creciente de que los productores abandonen las prácticas tradicionales de fermentación. Este cambio podría provocar la pérdida de la diversidad microbiana aquí descrita. Este trabajo proporciona un marco fundamental para desarrollar estrategias de conservación y gestión de estos hongos, destacando su valor más allá de la investigación básica. El sistema de fermentación de agave no solo es un reservorio de notable diversidad genética de levaduras, sino también un laboratorio natural único para obtener conocimientos sobre la interacción de factores genéticos, ecológicos y evolutivos que moldean la diferenciación poblacional y la introgresión entre especies.

MÉTODOS

Secuenciación del genoma e identificación de variantes. El ADN se purificó con el kit de purificación de ADN MasterPure, según las recomendaciones del fabricante, y se secuenció utilizando DNBSeq (2x150 pb; BGI, China). La calidad de las lecturas brutas se evaluó con fastp V0.20.0[47]. Para cada aislado, las lecturas filtradas se alinearon con BWA-MEM v0.7.4[48] con un genoma de referencia de especies concatenado que incluye *S. cerevisiae* S288C, *S. paradoxus* YPS138, *S. mikatae* IFO1815, *S. kudriavzevii* Cr85, *S. jurei* M1, *S. arboricola* H6, *S. uvarum* CBS7001, *S. eubayanus* FM1318, *K. marxianus* DMKU31042 y *P. kudriavzevii* CBS573. Las

lecturas duplicadas se marcaron con Picard 2.6.0[49], y se realizó una realineación local alrededor de las inserciones y deleciones y la identificación de variantes con GATK v4.1.1.0 [50]. La información sobre el equilibrio alélico se incorporó a los archivos VCF con el anotador de variantes GATK. Los genotipos llamados se filtraron para mantener solo los SNP bialélicos con una profundidad mínima de 5, una puntuación QUAL de al menos 30 y posiciones llamadas en al menos el 90 % de los aislados. Además, los SNP también se obtuvieron mapeando las lecturas filtradas sólo al genoma de referencia de *S. cerevisiae* para llamar variantes teniendo en cuenta los segmentos introgresados (Figura S3, Figura S4).

Análisis de escalamiento multidimensional. Se utilizaron datos de SNP a nivel genómico para el análisis de escalamiento multidimensional (MDS) con PLINK v1.9[47]. Se seleccionaron en total 1,262 aislados para proporcionar una visión global a nivel de especie, incluyendo 1,011 aislados de Peter *et al.* (2018) [6], 216 cepas recién secuenciadas en este estudio, 33 cepas de trabajos previos de secuenciación en el Neotrópico (Barbosa *et al.* 2016, Barbosa *et al.*, 2018)[16,41] y cuatro secuencias adicionales de linajes asociados a Sudamérica (SAM, Tellini *et al.*, 2024)[12]. Se utilizó el SRA Toolkit v3.0.0 para recuperar los archivos FASTQ correspondientes a muestras no secuenciadas en este manuscrito. Se excluyeron dos secuencias en la **Figura 1A** debido a redundancia con otras entradas detectadas con PLINK[47]. Sólo se consideraron SNPs nucleares, y se excluyeron variantes de baja frecuencia o raras de este análisis usando un umbral de tasa de llamadas perdidas > 1 %. Tras la aplicación de este filtro, se descartaron los aislados con genotipos ausentes > 10%. Los índices cuantitativos (componentes) de la variación genética para cada aislado se calcularon a partir de la proporción media de alelos a nivel genómico compartido entre cualquier par de individuos dentro de la muestra. Para el análisis MDS con representación balanceada entre clados (**Figura S2**), seguimos los mismos pasos descritos anteriormente, pero después seleccionamos aleatoriamente hasta cinco aislados de cada clado filogenético descritos en la siguiente sección.

Análisis filogenético. Los árboles de máxima verosimilitud se construyeron utilizando los genomas secuenciados en este estudio junto con cepas de referencia globales, con especial énfasis en los clados americanos. Especialmente, en las filogenias del material suplementario completas (**Figura S1**), incluimos 332 cepas distribuidas de la siguiente manera: las 216 cepas recién secuenciadas generadas en este estudio, 23 secuencias de Barbosa *et al.* (2016)[16], 10 aislados de Barbosa *et al.* (2018)[41], cuatro aislados sudamericanos (SAM) de Tellini *et al.* (2024)[12] y al menos un representante de cada clado definido por Peter *et al.* (2018) [6] (n=213 cepas). Para mejorar la resolución dentro de los linajes que contienen aislados asociados a agave, sobrerrepresentamos deliberadamente cepas de los clados Mixed Origin, Wine y North American Oak de Peter *et al.* (2018)[6]. Además, incorporamos 21 aislados de Alpechin de Pontes *et al.* (2019)[37], como referencia para determinar los efectos de altos niveles de introgresión. Las 487 cepas están indicadas en la columna *Used_in_Supplementary_Complete_Phylogenies* de **Datos Suplementarios S2**. Para la filogenia principal representada en la **Figura 1b**, usamos el mismo conjunto de cepas pero excluimos a los individuos mezclados ("admixed"). Sólo se retuvieron 332 cepas con > 85% de un solo componente genético predominante de acuerdo con el análisis ADMIXTURE para este árbol. Las 332 cepas están indicadas en la columna *Used_in_Main_Phylogeny* de los **Datos Suplementarios 2.**

Para generar las filogenias, se obtuvieron secuencias de haplotipos con vcf2phylip v2.3[48] de las variantes genómicas nucleares que se mapearon al subgenoma de *S. cerevisiae* del alineamiento al genoma multiespecífico concatenado, o cuando se indica así, directamente al genoma de referencia de *S. cerevisiae* para incluir variantes de introgresiones. Los árboles filogenéticos se infirieron usando IQTree 2.3.6 con el mejor modelo calculado con -m MFP y sumando el parámetro + ASC con 1000 bootstraps ultrarápidos y aLRT (aLRT mostrado para ramificaciones clave en **Figura S2**). Las filogenias también se reconstruyeron usando RAxML v8.2.12[49] (raxmlHPC-PTHREADS-AVX2) con el modelo GTR+GAMMA y 100 réplicas bootstrap. Dado que los resultados eran similares con ambos métodos, sólo se usaron árboles IQTree para las figuras del manuscrito. La visualización de

los árboles se realizó usando Microreact[50]. Para generar los árboles filogenéticos con un muestreo a la baja que tuviera el mismo número de cepas por clado (**Figura S2)**, seleccionamos aleatoriamente hasta cinco aislados de los clados filogenéticos incluidos en el conjunto de 332 genomas y construimos la filogenia con IQTree 2.3.6 con el parámetro ASC.

ADMIXTURE. Para el análisis ADMIXTURE, analizamos un conjunto de datos que comprende 466 cepas, centrado en los aislados asociados a agave recién secuenciados, como se indica en la columna *Used_in_ADMIXTURE* de **Datos Suplementarios 2**. Esto incluyó todas las cepas secuenciadas en el presente estudio (n = 216), junto con 213 genomas representativos de cepas de Peter *et al.* (2018)[6] asegurando la cobertura de todos los clados e incluyendo las 20 cepas que no estaban asignadas a algún clado en particular. Además, incorporamos 23 cepas de Barbosa *et al.* (2016)[16], 10 cepas de Barbosa *et al.* (2018)[41] y 4 cepas reportadas por Tellini *et al.* (2024)[6] que pertenecen al linaje SAM. Para mejorar la resolución dentro de los clados donde se agrupaban cepas asociadas a agave, sobrerrepresentamos intencionadamente los linajes de Mixed Origin, Wine y North American Oak de Peter *et al.* (2018)[6]. Para ejecutar ADMIXTURE 1.3.0[51], el VCF con 466 genomas fue curado usando PLINK 1.9[52] para remover el desequilibrio de ligamiento con parámetros previamente usados para *S. cerevisiae*[53] (tamaño de ventana de 50, deslizándose cada 5 nucleótidos y correlación de 0,5). Después de la curación se obtuvo un panel final de 618,253 SNVs. Se utilizaron diez semillas para ejecutar ADMIXTURE. El error de Validación Cruzada (CV) se obtuvo para modelos con K=2 a K=35. El CV más bajo se observó en K = 24 y por lo tanto éste fue el que se utilizó para la **Figura 3**. La visualización se hizo utilizando Pong 1.4.9 [54]. El paquete de R vegan (2.7-2)[55] se usó para realizar la prueba de Mantel Spearman.

Estimación de la diversidad genética. Para calcular la diversidad genética (π) y la *D* de Tajima, utilizamos VCFtools 0.1.14[56] y el VCF con variantes que mapeaban a *S. cerevisiae* tras mapear a la referencia concatenada y filtrar SNPs bialélicos de alta calidad con menos del 10% de datos faltantes. Se estimó la heterocigosidad por

variante usando BCFtools 1.9[57] sobre los VCFs de las cepas individuales para calcular la proporción entre el número de variantes heterocigotas y el número de variantes.

Análisis de las introgresiones. Para identificar genes introgresados, obtuvimos las regiones correspondientes al genoma de *S. paradoxus* cuando se realizaba el alineamiento con la referencia concatenada. Un gen se consideraba introgresado si más de la mitad tenía una profundidad de cobertura superior al 25% de la profundidad mediana de toda la referencia. Además, tenía que ser el ortólogo con mayor profundidad o, si no, su profundidad debía ser al menos el 25% de la de su ortólogo en el subgenoma de *S. cerevisiae*. También era requisito una diferencia mínima del 5% a nivel de nucleótidos entre los ortólogos de *S. cerevisiae* y *S. paradoxus*. Un gen introgresado se consideraba heterocigoto si la relación de la cobertura de profundidad entre los dos subgenomas en el mapeo competitivo era entre 0,25 y 4.

Tras superar estos umbrales de mapeo a nivel de gen, agrupamos genes consecutivos en bloques según la anotación[58] y evaluamos la posición filogenética de cada bloque respecto a un panel de dos genomas de *S. cerevisiae* (Wine ERS1082532 y Taiwanese ERS1082750) y cinco de *S. paradoxus* (*SpA* SRR4074385, *SpC* SRR7500262, *SpB* SRR4074411, *SpB_Bra* SRR4074412, *SpB_MxAgave* YMX005537) con IQtree. Usando ape 5.0[59], sólo conservamos como verdaderas introgresiones aquellos bloques que se agrupaban con *S. paradoxus* en la filogenia.

El Gene Ontology Enrichment se realizó utilizando la herramienta GO Term Finder de la base de datos genómica de *Saccharomyces*. Se usaron genes introgresados presentes en al menos 75% de las cepas dentro de cada clado como conjunto de consultas, sin especificar el conjunto de fondo. Utilizando los parámetros predeterminados, no se encontraron términos enriquecidos o eran demasiado ambiguos para proporcionar conocimientos biológicos relevantes.

Una vez determinados los bloques introgresados para inferir el origen de las introgresiones de *S. cerevisiae*, mapeamos el panel completo de genomas de *S. paradoxus* de López-Gallegos *et al.* (2025)[22] al genoma de referencia concatenado que incluye tanto *S. cerevisiae* como *S. paradoxus*. Sólo conservamos el subgenoma de *S. paradoxus*. El VCF resultante se filtró para retener solo las regiones previamente identificadas como introgradadas desde *S. paradoxus* hacia cepas de *S. cerevisiae*. Utilizando este VCF, que contiene regiones introgradadas en *S. cerevisiae* y las variantes correspondientes de *S. paradoxus*, se calcularon valores pareados de identidad por estado (IBS) mediante PLINK v1.9.9[51]. Para cada región introgradada, se asignó su origen probable identificando el clado de S. paradoxus que contiene la cepa con el valor más alto de identidad por estado (IBS). Cuando las regiones introgradadas mostraron una similitud igualmente alta (IBS) con cepas de múltiples linajes de S. paradoxus, no se asignó ningún origen. Reportamos el origen de las regiones introgradadas según el clado más cercano basado en la similitud de IBS, ya que este enfoque aprovecha el conjunto de datos más extenso, que incluye todas las cepas de *S. paradoxus* de López-Gallegos et al 2025[60], y proporciona una mayor resolución para asignar el origen de un mayor número de introgresiones. Esta asignación es concordante con los resultados obtenidos mediante métricas alternativas, incluida la identificación de hermanos filogenéticos y la asignación al clado de la cepa con la menor distancia filogenética (Datos Suplementarios 4, columnas *ClosestClade_by_IBS_Similarity, Siblings_in_Phylogeny y Strain_with_LowestDistance_in_Phylogeny*). Todos los genes dentro de un bloque dado se consideraban del mismo origen.

Generación de mapas. Los mapas se generaron en R utilizando ggplot2 4.0.0[61]. Los datos de líneas de costa y límites políticos a nivel mundial se obtuvieron del conjunto de datos Natural Earth mediante el paquete rnaturalearth 1.1.0 [62]

DISPONIBILIDAD DE DATOS

Los datos de secuenciación genómica generados en este estudio se han depositado en el NCBI SRA bajo el BioProject PRJNA1138754 [https://www.ncbi.nlm.nih.gov/bioproject/?term=PRJNA1138754]. Los números de

accesión de cada genoma utilizado en este estudio, incluidos aquellos previamente secuenciados, se proporcionan en los Datos Suplementarios 1 y 2. Esta traducción al español del artículo, está incluida en los Datos Suplementarios 5. Los datos fuente se proporcionan junto con este artículo.

DISPONIBILIDAD DE CÓDIGO

Scripts personalizados. Los scripts para identificar genes introgresados, determinar su origen y generar las cifras presentadas en este estudio están disponibles en: https://github.com/GELab-LIIGH/ScerYeastGenomesMx2024[63]

REFERENCIAS

Las referencias en este documento se encuentran numeradas conforme a la lista de Referencias de la versión original en inglés.

1. Liti, G. The Natural History of Model Organisms: The fascinating and secret wild life of the budding yeast *S. cerevisiae*. *eLife* https://elifesciences.org/articles/05835 (2015) doi:10.7554/eLife.05835.
2. Marcet-Houben, M. & Gabaldón, T. Beyond the Whole-Genome Duplication: Phylogenetic Evidence for an Ancient Interspecies Hybridization in the Baker's Yeast Lineage. *PLOS Biol.* **13**, e1002220 (2015).
3. Alsammar, H. & Delneri, D. An update on the diversity, ecology and biogeography of the *Saccharomyces* genus. *FEMS Yeast Res.* **20**, foaa013 (2020).
4. Bendixsen, D. P., Gettle, N., Gilchrist, C., Zhang, Z. & Stelkens, R. Genomic Evidence of an Ancient East Asian Divergence Event in Wild *Saccharomyces cerevisiae*. *Genome Biol. Evol.* **13**, evab001 (2021).
5. Wang, M. *et al.* Annotation of 2,507 *Saccharomyces cerevisiae* genomes. *Microbiol. Spectr.* **12**, e03582-23 (2024).
6. Peter, J. *et al.* Genome evolution across 1,011 *Saccharomyces cerevisiae* isolates. *Nature* **556**, 339–344 (2018).
7. Pontes, A., Hutzler, M., Brito, P. H. & Sampaio, J. P. Revisiting the Taxonomic Synonyms and Populations of *Saccharomyces cerevisiae*—Phylogeny, Phenotypes, Ecology and Domestication. *Microorganisms* **8**, 903 (2020).
8. Loegler, V., Friedrich, A. & Schacherer, J. Overview of the *Saccharomyces cerevisiae* population structure through the lens of 3,034 genomes. 2024.09.16.613241 Preprint at https://doi.org/10.1101/2024.09.16.613241 (2024).
9. Duan, S.-F. *et al.* The origin and adaptive evolution of domesticated populations of yeast from Far East Asia. *Nat. Commun.* **9**, 2690 (2018).
10. Strope, P. K. *et al.* The 100-genomes strains, an *S. cerevisiae* resource that illuminates its natural phenotypic and genotypic variation and emergence as an opportunistic pathogen. *Genome Res.* **25**, 762–774 (2015).

11. Clark, A., Dunham, M. J. & Akey, J. M. The genomic landscape of *Saccharomyces paradoxus* introgression in geographically diverse *Saccharomyces cerevisiae* strains. 2022.08.01.502362 Preprint at https://doi.org/10.1101/2022.08.01.502362 (2022).
12. Tellini, N. *et al.* Ancient and recent origins of shared polymorphisms in yeast. *Nat. Ecol. Evol.* **8**, 761–776 (2024).
13. Boynton, P. J. & Greig, D. The ecology and evolution of non-domesticated *Saccharomyces* species. *Yeast Chichester Engl.* **31**, 449–462 (2014).
14. Shen, X.-X. *et al.* Tempo and Mode of Genome Evolution in the Budding Yeast Subphylum. *Cell* **175**, 1533-1545.e20 (2018).
15. Morrone, J. J. Biogeographical regionalisation of the Neotropical region. *Zootaxa* **3782**, 1–110 (2014).
16. Barbosa, R. *et al.* Evidence of Natural Hybridization in Brazilian Wild Lineages of *Saccharomyces cerevisiae*. *Genome Biol. Evol.* **8**, 317–329 (2016).
17. De Chiara, M. *et al.* Domestication reprogrammed the budding yeast life cycle. *Nat. Ecol. Evol.* **6**, 448–460 (2022).
18. D'Angiolo, M. *et al.* A yeast living ancestor reveals the origin of genomic introgressions. *Nature* **587**, 420–425 (2020).
19. Peris, D. *et al.* Macroevolutionary diversity of traits and genomes in the model yeast genus *Saccharomyces*. *Nat. Commun.* **14**, 690 (2023).
20. Gallegos-Casillas, P. *et al.* Yeast diversity in open agave fermentations across Mexico. *Yeast* **41**, 35–51 (2024).
21. Chai, C. *et al.* Three novel Ascomycota (Saccharomycetes, Saccharomycetales) yeast species derived from the traditional Mexican alcoholic beverage Pulque. *MycoKeys* **109**, 187–206 (2024).
22. López-Gallegos, C. *et al.* Ecological divergence of sympatric *Saccharomyces* species across wild and fermentative environments in the neotropics. 2025.05.31.656962 Preprint at https://doi.org/10.1101/2025.05.31.656962 (2025).
23. Colón-González, M. *et al.* Thriving in Adversity: Yeasts in the Agave Fermentation Environment. *Yeast* **42**, 16–30 https://doi.org/10.1002/yea.3989 (2025).
24. Mittermeier, R. A. & Mittermeier, C. G. *Megadiversity: Earths Biologically Wealthiest Nations*. (CEMEX, 2005).
25. Lachance, M.-A. Yeast communities in a natural tequila fermentation. *Antonie Van Leeuwenhoek* **68**, 151–160 (1995).
26. Verdugo Valdez, A. *et al.* Yeast communities associated with artisanal mezcal fermentations from Agave salmiana. *Antonie Van Leeuwenhoek* **100**, 497–506 (2011).
27. Álvarez-Ainza, M. L., Zamora-Quiñonez, K. A., Moreno-Ibarra, G. M. & Acedo-Félix, E. Genomic Diversity of Saccharomyces cerevisiae Yeasts Associated with Alcoholic Fermentation of Bacanora Produced by Artisanal Methods. *Appl. Biochem. Biotechnol.* **175**, 2668–2676 (2015).
28. Kirchmayr, M. R. *et al.* Impact of environmental conditions and process modifications on microbial diversity, fermentation efficiency and chemical profile during the fermentation of *Mezcal* in Oaxaca. *LWT - Food Sci. Technol.* **79**, 160–169 (2017).
29. Bruman, H. J. *Alcohol in Ancient Mexico*. (University of Utah Press, 2000).

30. Arellano-Plaza, M., Paez-Lerma, J. B., Soto-Cruz, N. O., Kirchmayr, M. R. & Gschaedler Mathis, A. Mezcal Production in Mexico: Between Tradition and Commercial Exploitation. *Front. Sustain. Food Syst.* **6**, (2022).
31. Colón-González, M. *et al.* Thriving in Adversity: Yeasts in the Agave Fermentation Environment. Yeast. https://doi.org/10.1002/yea.3989 (2025).
32. Padilla-Camberos, E., Pinal-Zuazo, L. & Alvarez de la Cuadra Jacob, J. Catálogo de la colección de cultivos microbianos. (1994).
33. Kirchmayr, M. R. *et al. Manual para la estandarización de los procesos de producción del mezcal guerrerense*. vol. 1 (Centro de Investigación y Asistencia en Tecnología y Diseño del Estado de Jalisco A.C., Guadalajara, Jalisco. México, 2014).
34. Quezada, R., Gschaedler, A. & Kirchmayr, M. Characterization of microbial population dynamics associated with different juices of *Agave tequilana*. in *Sustainable and Integrated use of Agave* vol. 2016 2016 (2016, Zapopan, Jalisco, Mexico, 2016).
35. Sampaio, J. P. & Pontes, A. Yeast domestication. *Curr. Biol.* **35**, R575–R586 (2025).
36. Rzedowski, J. *Vegetación de México*. (1978).
37. Mastretta-Yanes, A., Moreno-Letelier, A., Piñero, D., Jorgensen, T. H. & Emerson, B. C. Biodiversity in the Mexican highlands and the interaction of geology, geography and climate within the Trans-Mexican Volcanic Belt. https://doi.org/10.1111/jbi.12546 (2015) doi:10.1111/jbi.12546.
38. Pontes, A., Čadež, N., Gonçalves, P. & Sampaio, J. P. A Quasi-Domesticate Relic Hybrid Population of *Saccharomyces cerevisiae* × *S. paradoxus* Adapted to Olive Brine. *Front. Genet.* **10**, (2019).
39. Diop, A., Torrance, E. L., Stott, C. M. & Bobay, L.-M. Gene flow and introgression are pervasive forces shaping the evolution of bacterial species. *Genome Biol.* **23**, 239 (2022).
40. Edelman, N. B. & Mallet, J. Prevalence and Adaptive Impact of Introgression. *Annu. Rev. Genet.* **55**, 265–283 (2021).
41. Barbosa, R. *et al.* Multiple Rounds of Artificial Selection Promote Microbe Secondary Domestication—The Case of Cachaça Yeasts. *Genome Biol. Evol.* **10**, 1939–1955 (2018).
42. Jacobus, A. P. *et al.* Comparative Genomics Supports That Brazilian Bioethanol *Saccharomyces cerevisiae* Comprise a Unified Group of Domesticated Strains Related to Cachaça Spirit Yeasts. *Front. Microbiol.* **12**, (2021).
43. O'Donnell, S. *et al.* 142 telomere-to-telomere assemblies reveal the genome structural landscape in *Saccharomyces cerevisiae*. 2022.10.04.510633 Preprint at https://doi.org/10.1101/2022.10.04.510633 (2022).
44. Demogines, A., Wong, A., Aquadro, C. & Alani, E. Incompatibilities Involving Yeast Mismatch Repair Genes: A Role for Genetic Modifiers and Implications for Disease Penetrance and Variation in Genomic Mutation Rates. *PLOS Genet.* **4**, e1000103 (2008).
45. Colunga García Marín, S. P. *et al. Los agaves y las prácticas mesoamericanas de aprovechamiento, manejo y domesticación*. (2017).
46. Jara-Servin, A. *et al.* Microbial Communities in Agave Fermentations Vary by Local Biogeographic Regions. *Environ. Microbiol. Rep.* **17**, e70057 (2025).
47. Purcell, S. *et al.* PLINK: A Tool Set for Whole-Genome Association and Population-Based Linkage Analyses. *Am. J. Hum. Genet.* **81**, 559–575 (2007).

48. Ortiz, E. M. vcf2phylip v2.0: convert a VCF matrix into several matrix formats for phylogenetic analysis. zenodo https://doi.org/DOI:10.5281/zenodo.2540861.
49. Stamatakis, A. RAxML version 8: a tool for phylogenetic analysis and post-analysis of large phylogenies. *Bioinformatics* **30**, 1312–1313 (2014).
50. Argimón, S. *et al.* Microreact: visualizing and sharing data for genomic epidemiology and phylogeography. *Microb. Genomics* **2**, e000093 (2016).
51. Alexander, D. H., Novembre, J. & Lange, K. Fast model-based estimation of ancestry in unrelated individuals. *Genome Res.* **19**, 1655–1664 (2009).
52. Purcell, S. & Chang, C. PLINK 1.9. www.cog-genomics.org/plink/1.9/.
53. Gallone, B. *et al.* Domestication and Divergence of *Saccharomyces cerevisiae* Beer Yeasts. *Cell* **166**, 1397-1410.e16 (2016).
54. Behr, A. A., Liu, K. Z., Liu-Fang, G., Nakka, P. & Ramachandran, S. pong: fast analysis and visualization of latent clusters in population genetic data. *Bioinformatics* **32**, 2817–2823 (2016).
55. Oksanen, J. *et al.* vegan: Community Ecology Package. (2025).
56. Danecek, P. *et al.* The variant call format and VCFtools. *Bioinformatics* **27**, 2156–2158 (2011).
57. Danecek, P. *et al.* Twelve years of SAMtools and BCFtools. *GigaScience* **10**, giab008 (2021).
58. Yue, J.-X. *et al.* Contrasting evolutionary genome dynamics between domesticated and wild yeasts. *Nat. Genet.* **49**, 913–924 (2017).
59. Paradis, E. & Schliep, K. ape 5.0: an environment for modern phylogenetics and evolutionary analyses in R. *Bioinformatics* **35**, 526–528 (2019).
60. Lopez-Gallegos, C. & Aguirre-Dugua, X. Contrasting distribution of *Saccharomyces cerevisiae* and *S. paradoxu*s among open fermentations, vectors, reservoirs and natural sites. *Biorxiv* (2025).
61. ggplot2: Elegant Graphics for Data Analysis (3e). https://ggplot2-book.org/.
62. World Map Data from Natural Earth. https://docs.ropensci.org/rnaturalearth/.
63. Avelar-Rivas, J. A. *et al.* GELab-LIIGH/ScerYeastGenomesMx2024: Avelar-Rivas2024. Zenodo https://doi.org/10.5281/zenodo.17970285 (2025).

AGRADECIMIENTOS

Agradecemos a Luis Aguilar (LIIGH-LAVIS, UNAM), Porfirio Gallegos (CINVESTAV), Aarón de Luna (LIIGH), Alejandra Castillo (LIIGH), Carina Uribe (LIIGH), Maritrini Colón-González (LIIGH) y Jair García (LIIGH) por su asistencia técnica. Agradecemos a Bernard Dujon y María Ávila por la lectura crítica del manuscrito. Agradecemos a Gianni Liti, Luis Delaye, Diego Ortega, Marcela Sandoval, Nicolò Tellini y Alicia Mastretta-Yanes por sus útiles debates, y a Manuel R. Kirchmayr (CIATEJ), Anne Gschaedler (CIATEJ), Maritza Álvarez (CIAD) y Marc-André Lachance por compartir sus opiniones. Estamos profundamente agradecidos con todos los productores de destilados y bebidas fermentadas de agave en México que amablemente participaron en este trabajo proporcionando acceso a muestras de fermentación y compartiendo sus conocimientos. Detalles sobre los agradecimientos relativos a la ayuda en el trabajo de campo y el muestreo del consorcio YeastGenomesMx pueden encontrarse en Gallegos Casillas *et al.* (2024) [20]. Este trabajo fue financiado por la Secretaría de Ciencia, Humanidades, Tecnología e Innovación de México (Secihti) proyectos FORDECYT-PRONACES/103000/2020, CF-2023-G-695, CBF-2025-G-838, Fondo de Investigación y Desarrollo Tecnológico del Cinvestav (SEP-CINVESTAV/023), UNAM-PAPIIT (proyectos IN209021, IN230420 y IN212524), UNAM-UI Fondos Semilla 2023 y el BBSRC del Reino Unido bajo la convocatoria Global Challenges Research Fund (GCRF) para una Creciente Capacidad de Investigación a través del Fondo de Innovación CABANA (BB/P027849/1). EM fue financiado por Secihti para una estancia sabática (111/I0200/2024). LFG-O fue investigador postdoctoral financiado por Secihti (4133922); IS es estudiante de doctorado en la UNAM con una beca de posgrado de Secihti. JAUA y CL-G fueron estudiantes de maestría con una beca de posgrado de Secihti.

CONTRIBUCIONES DE LOS AUTORES

Conceptualización: EM, ADL, LM; Metodología: JAA-R, IS, LFG-O, JAUA, CL-G, XA-D; Curación de datos: JAA-R, IS and LFG-O; Investigación: CL-G; Análisis

formal: JAA-R, IS, LFG-O, JAUA, CL-G, XA-D; Visualización: JAA-R and IS; Adquisición de financiamiento: EM, ADL, LM. Supervisión: EM, ADL, LM. Guion—borrador original: JAA-R, EM, ADL, LM. Escritura—revisión y edición: JAA-R, IS, LFG-O, JAUA, CL-G, XA-D, EM, ADL, LM.

DECLARACIÓN DE CONFLICTO DE INTERÉS

Los autores declaran no tener conflicto de interés.

FIGURAS

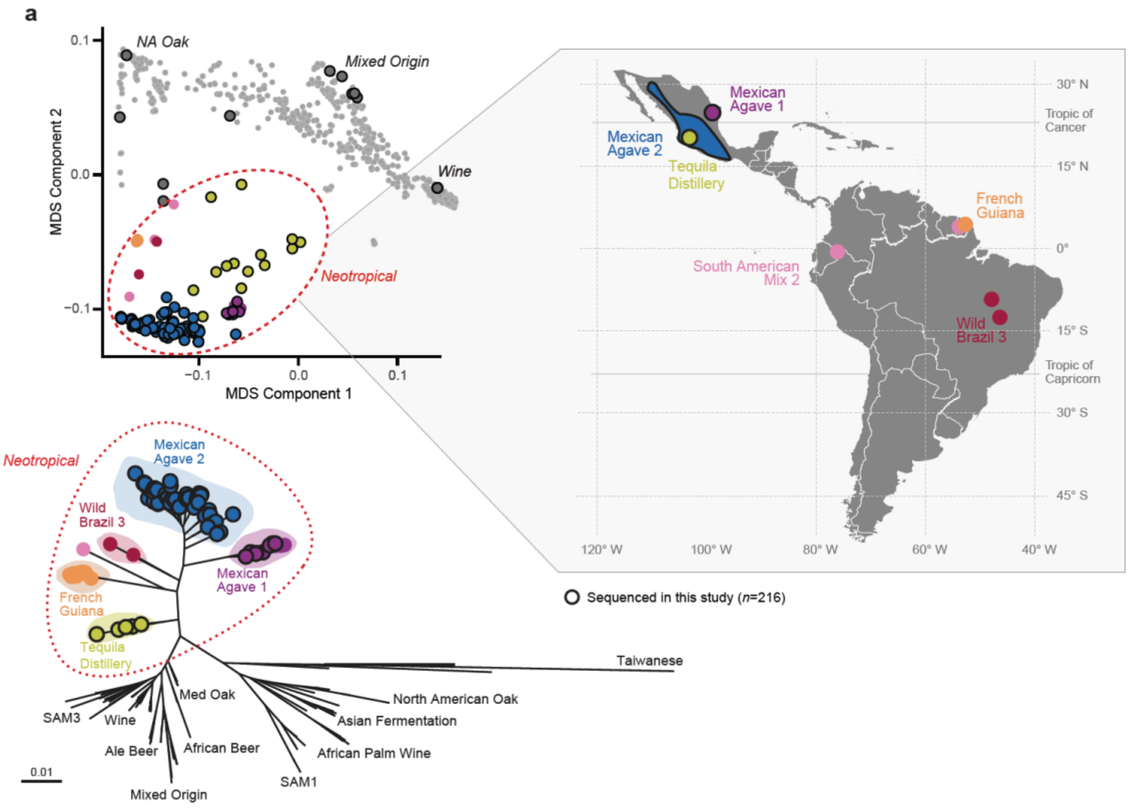

Figura 1. Un linaje novedoso divergente agrupa la mayor parte de la diversidad de levaduras provenientes de fermentaciones espontáneas abiertas de agave. a Análisis MDS de 1,262 genomas de cepas de todo el mundo (173,331 sitios informativos). Los puntos grandes son cepas de América tropical coloreadas según su clado: "Mexican Agave 1" (morado), "Mexican Agave 2" (azul), "French Guiana" (naranja), "Tequila Distillery" (amarillo), "Wild Brazil 3" (carmesí) y "South American Mix 2" (SAM2, rosa). Pequeños puntos grises indican aislados de otras partes del mundo, con las referencias de dónde se ubican cepas de los clados Wine, Mixed Origin y North American Oak (NA Oak). Los contornos sólidos negros señalan cepas secuenciadas en este estudio (n=216). Los círculos grises con un contorno sólido indican los pocos aislados de fermentación de agave que no se agruparon con otras cepas neotropicales. **b** Árbol filogenético de máxima verosimilitud construido con 694,264 SNPs en 332 cepas, incluyendo un subconjunto de genomas de referencia representativos de cepas de todo el mundo y excluyendo cepas mezcladas según el análisis ADMIXTURE (Figura 3a, véase Métodos). Para mayor claridad, sólo se etiquetan los clados clave, aunque todos los linajes relevantes de la especie se incluyeron en la filogenia (véase la Datos Suplementarios S2). Los nombres de clados son según Tellini N, *et al.* (2024)[12]; para los nombres originales de otros clados neotropicales, véanse Barbosa *et al.* (2016)[16], Barbosa *et al.* (2018)[41] y Peter *et al.* (2018)[6].

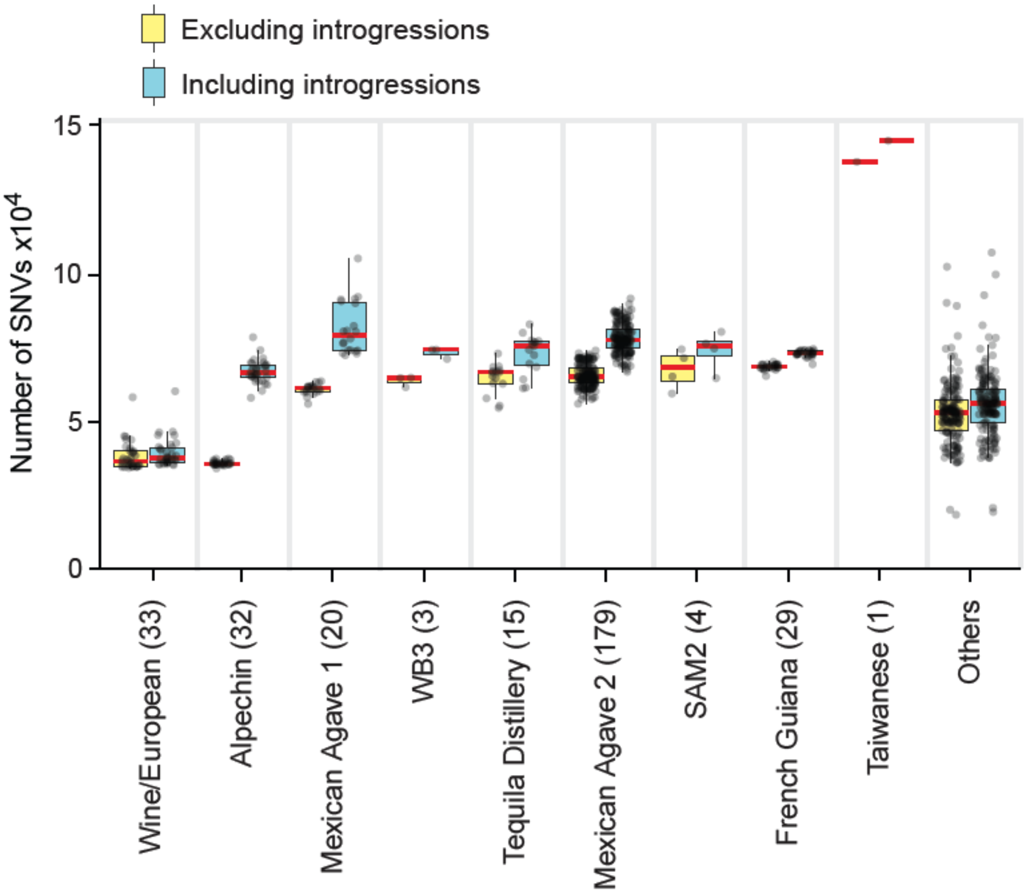

Figura 2. La divergencia del grupo Neotropical no se debe sólo a introgresiones. Los diagramas de caja muestran los percentiles 25 y 75 y los bigotes que se extienden hasta los valores mínimos y máximos dentro de 1,5 veces el rango intercuartil. Los puntos representan observaciones individuales de cada clado. Las líneas horizontales rojas indican las medianas de los conteos de SNV para 487 cepas agrupadas por clado filogenético. Los SNVs se llamaban ya sea desde alineamientos al genoma de referencia de *S. cerevisiae* (azul, incluyendo regiones introgresadas) o al subgenoma de *S. cerevisiae* a partir del alineamiento a una referencia multiespecie concatenada (amarillo, excluyendo regiones introgresadas) (véase Métodos). WB3, Wild Brazil 3; SAM2, South American Mix 2.

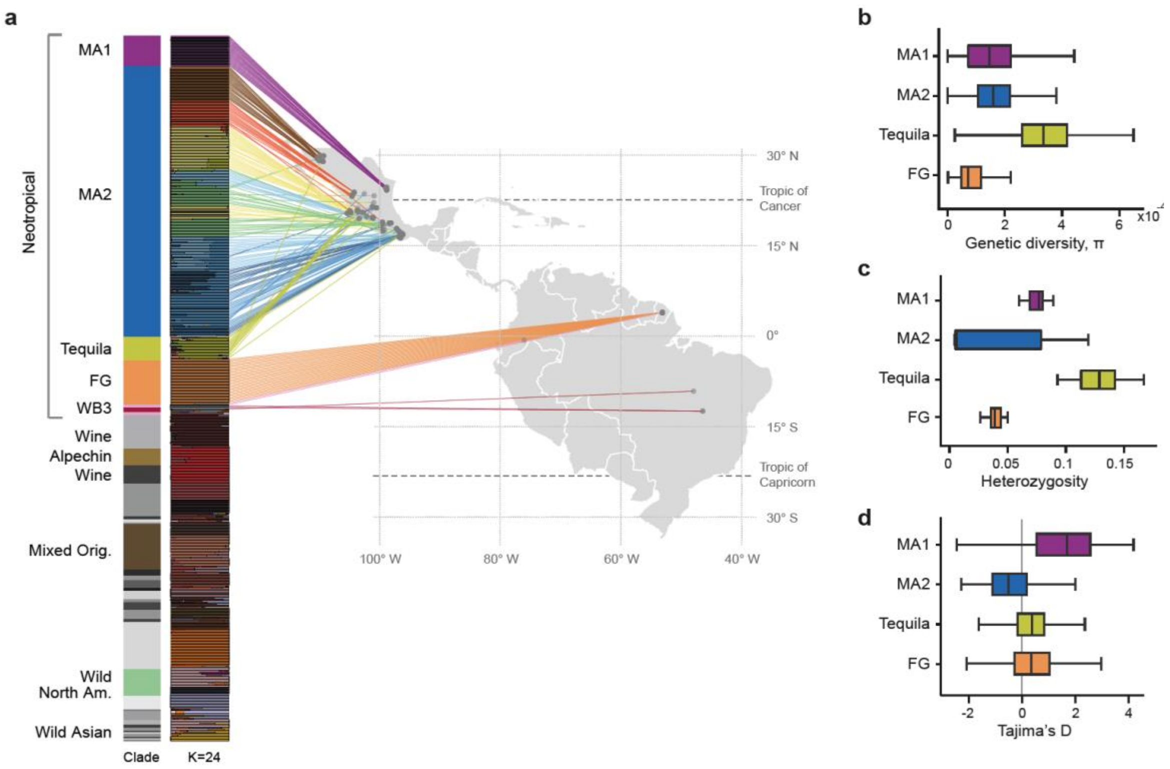

Figura 3. Las levaduras del Neotrópico muestran una estructura poblacional correlacionada con la geografía. a Estructura poblacional de cepas del Neotrópico y otras regiones del mundo como referencia en K=24 producida con ADMIXTURE (n=466); K=24 fue el K con el menor error de validación cruzada (Figura Suplementaria S5); las cepas están ordenadas según los clados filogenéticos (primera banda desde la izquierda). A la derecha, la ubicación geográfica de cada cepa aparece en el mapas de México y parte de Sudamérica que abarca Brasil, Ecuador y Guayana Francesa. Los colores de las líneas que conectan las cepas con la ubicación geográfica son los mismos que el componente genético más prevalente de la cepa según ADMIXTURE (segunda banda desde la izquierda). MA1, Mexican Agave 1; MA2, Mexican Agave 2; FG, French Guiana; WB3, Wild Brazil 3. **b** Diversidad genética, π (arriba), heterocigosidad (centro) y *D* de Tajima (abajo) de los clados que pertenecen al grupo Neotropical. Los diagramas de caja muestran los percentiles 25 y 75, con líneas verticales que indican las medianas de todos los valores. Los bigotes se extienden hasta los valores mínimo y máximo dentro de 1,5 veces el rango intercuartil. Los más extremos no se muestran explícitamente. Solo se muestran los clados con más de 10 secuencias disponibles. El número de cepas consideradas para cada clado fue de n = 20 para MA1; n = 178 para MA2; n = 15 para Tequila; y n = 29 para FG; solo se muestran los clados con más de 10 secuencias disponibles. Para los análisis de diversidad genética y el estadístico D de Tajima, el número de ventanas de 10 kb utilizadas para los diagramas de caja fue de n = 1,135 para MA1, n = 1,145 para MA2, n = 1,144 para Tequila y n = 1,139 para FG. Los datos fuente se proporcionan como un archivo de Datos Fuente.

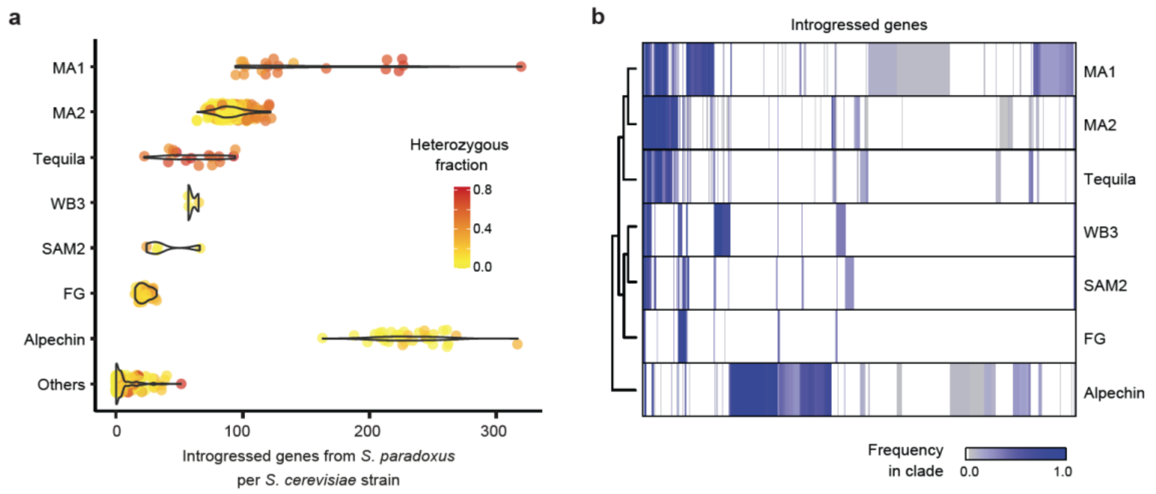

Figura 4. Diferentes patrones de introgresiones en los clados del Neotrópico. a Distribución del número de genes introgresados por cepa en cada grupo. Las cepas de Alpechin se incluyen como referencia de una población independiente y altamente introgresada. El color de cada punto, tal como se muestra en la escala de colores, indica la fracción de genes introgresados que son heterocigotos en cada cepa, lo que significa que están presentes tanto los alelos de *S. cerevisiae* como los de *S. paradoxus*. **b** Agrupamiento de los genes introgresados del grupo Neotropical y el clado de Alpechin (n = 1,226 en total, 832 en clados neotropicales). Los colores indican la fracción de cepas de cada clado que tenían el gen introgresado, tal y como se representa en la leyenda. MA1, Mexican Agave 1; MA2, Mexican Agave 2; WB3, Wild Brazil 3; SAM2, South American Mix 2; FG, French Guiana.

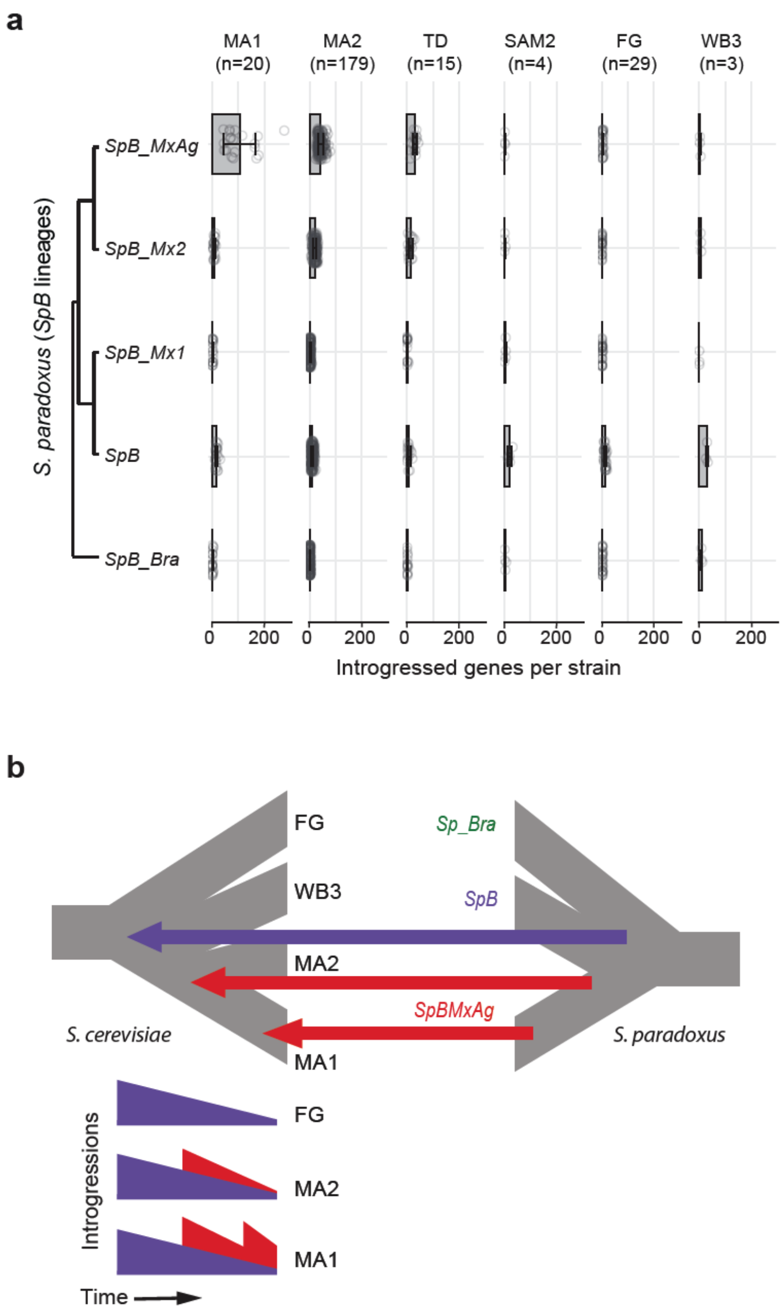

Figura 5. Pulsos de introgresión múltiples de dos linajes de *S. paradoxus* en el grupo Neotropical de *S. cerevisiae*. a Número de genes introgresados de cada linaje de *S. paradoxus* en cada clado neotropical de *S. cerevisiae*. Cada círculo representa un genoma, la barra muestra la media y las barras de error son una desviación estándar. **b** Esquema del modelo que muestra múltiples pulsos de introgresión (flechas moradas y rojas) como el escenario evolutivo más plausible para explicar los patrones observados sobre el número, heterocigosidad, longitud y origen de los bloques introgresados de *S. paradoxus*. MA1, Mexican Agave 1; MA2, Mexican Agave 2; TD, Tequila Distillery; SAM2, South American Mix 2; FG, French Guiana; WB3, Wild Brazil 3.

FIGURAS SUPLEMENTARIAS

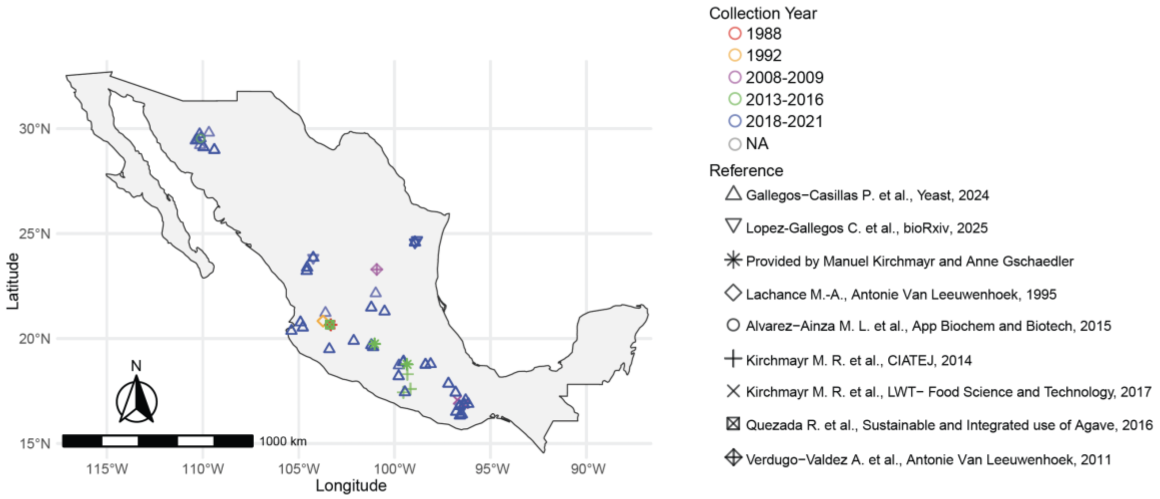

Figura Suplementaria S1. Origen de las 216 cepas secuenciadas en el presente estudio, obtenidas a partir de diferentes esfuerzos de muestreo en el país entre los años 1988 y 2021[1-9]. La forma de cada símbolo representa la referencia del muestreo, mientras que su color indica el año en que se recolectó el aislado.

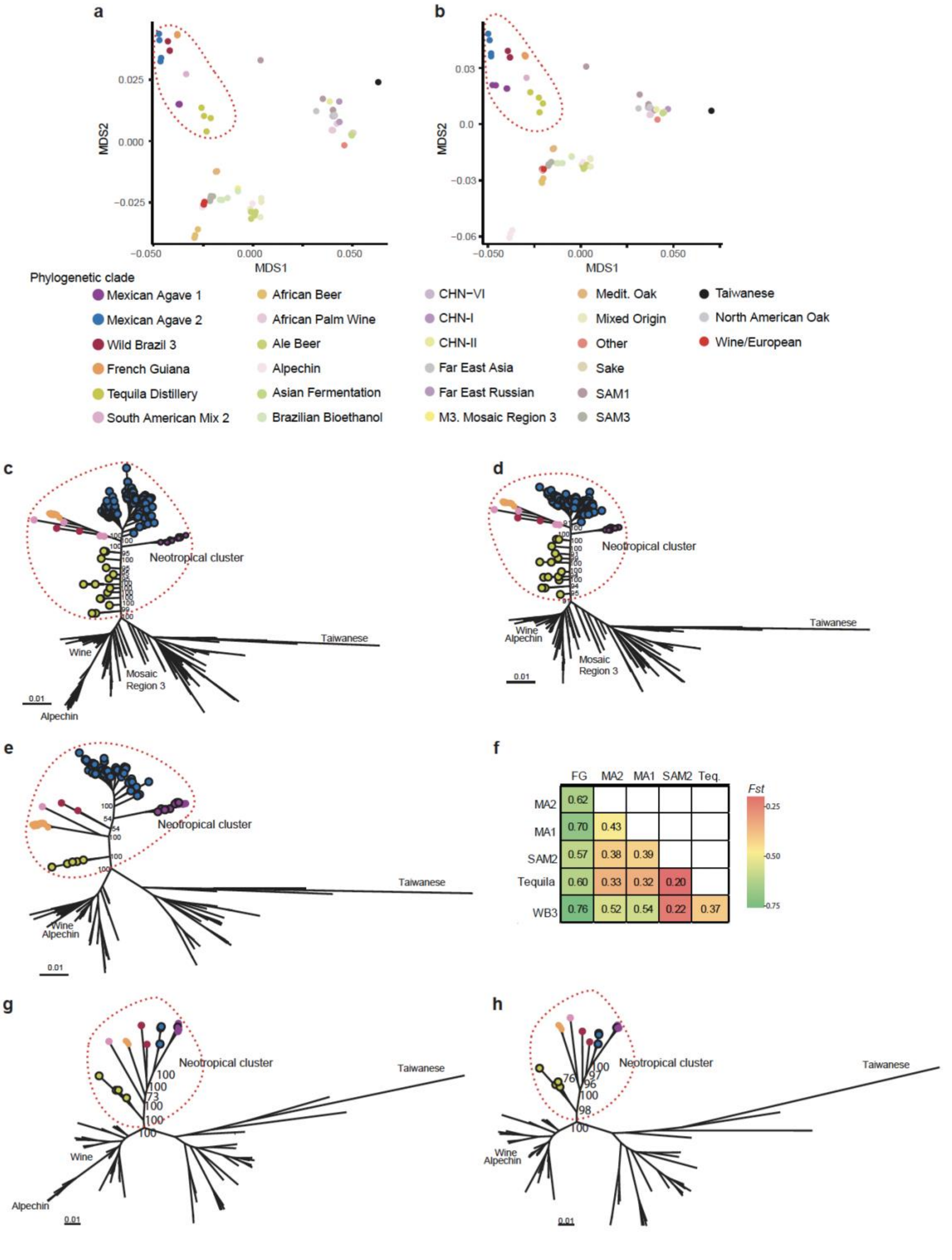

Figura Suplementaria S2. El clúster neotropical permanece coherente independientemente de la profundidad de muestreo o de la presencia de introgresiones. a Análisis de Escalamiento Multidimensional (MDS) de un conjunto de datos submuestreado que incluye hasta cinco cepas por clado, considerando las introgresiones. El gráfico MDS se generó utilizando SNPs llamados a partir de alineamientos contra la referencia de *S. cerevisiae*, que retiene SNPs de regiones introgresadas.

b Gráfico MDS generado utilizando SNPs llamados a partir de alineamientos contra una referencia concatenada que conserva únicamente los SNPs presentes en el subgenoma nuclear de *S. cerevisiae*, excluyendo así los SNPs provenientes de introgresiones. **c** Filogenia de máxima verosimilitud con un conjunto de 486 genomas, incluyendo SNVs dentro de las introgresiones (1,177,709 SNPs). **d** Filogenia de máxima verosimilitud con el mismo conjunto de 486 cepas, pero excluyendo las introgresiones (1,002,285 SNPs). **e** Filogenia de máxima verosimilitud de 332 cepas, como se describe en la Figura 1B del texto principal. **f**, Diferenciación genética entre los grupos neotropicales, utilizando F_{ST} ponderado.**g–h** Filogenias de máxima verosimilitud inferidas a partir de un conjunto submuestreado de genomas (hasta cinco cepas por clado), incluyendo (**g**) o excluyendo (**h**) las regiones introgresadas. Todas las filogenias se generaron con IQ-TREE utilizando corrección por sesgo de aserción, con soporte aLRT indicado para nodos clave. Los puntos coloreados en las puntas de las ramas indican el origen de las cepas de *S. cerevisiae* del clúster neotropical: Mexican Agave 1 (morado), Mexican Agave 2 (azul), French Guiana (naranja), Tequila Distillery (amarillo), Wild Brazil 3 (carmín), South American Mix 2 (rosa); las cepas de Vino, Alpechín, Región Mosaico y Taiwán se muestran como referencia.

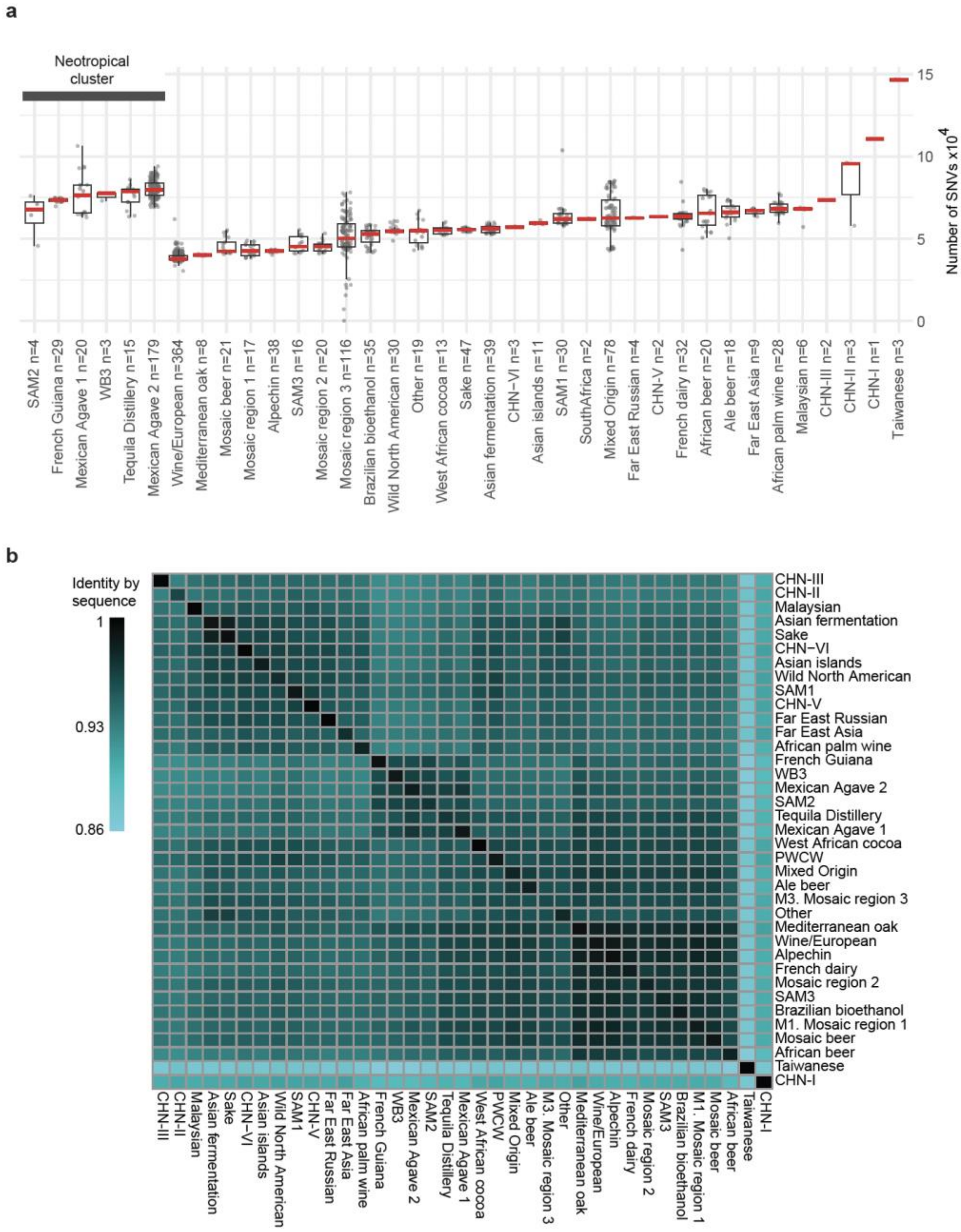

Figura Suplementaria S3. Las cepas neotropicales agrupadas en clados muestran un alto número de SNVs en comparación con la diversidad mundial. **a** Los diagramas de caja muestran los percentiles 25 y 75, las líneas horizontales indican las medianas de los conteos de SNVs de las cepas que representan la diversidad mundial agrupada por clado. Los clados neotropicales están en el extremo izquierdo y el resto está ordenado por la mediana de SNVs mostrada en rojo. Las variantes de las cepas recién secuenciadas se fusionaron con el VCF de Peter *et al.*, 2018[10], y el número de variantes se calculó usando bcftools 1.9. **b** Identidad mediana por secuencia entre clados usando el VCF con los SNVs de los 487 genomas usados para la reconstrucción filogenética completa.

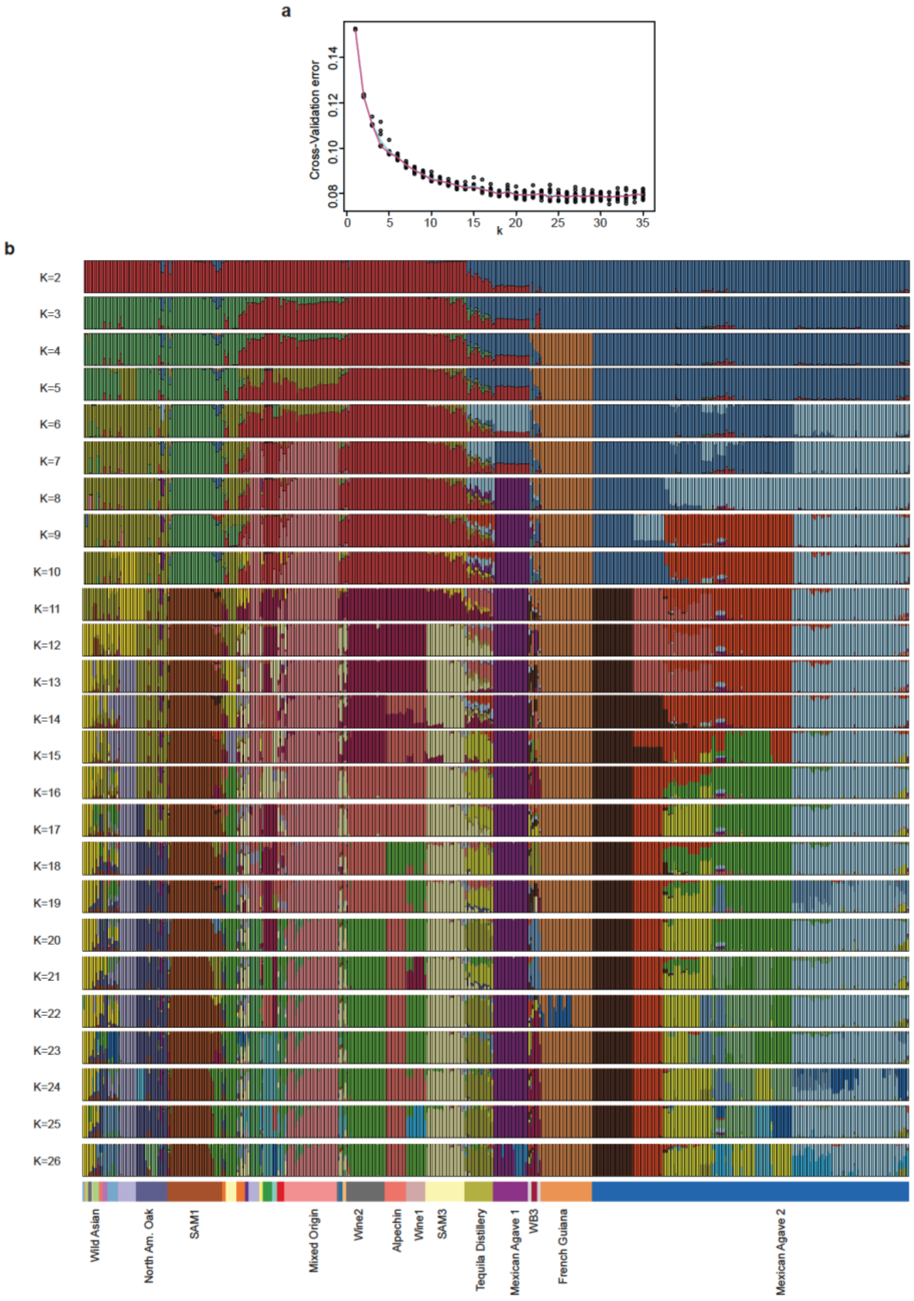

Figura Suplementaria S4. Análisis de la estructura poblacional con ADMIXTURE. (A) Análisis de error de validación cruzada, donde cada punto es una de diez réplicas para diferentes valores de K. Las líneas cian y magenta son la media y la mediana, respectivamente. (B) Análisis de la estructura poblacional de mezcla con los errores de validación cruzada más bajos (K=3 a K=26). Los grupos denominados South American Mix (Tellini *et al.* 2024)[11] están integrados como sigue: SAM1 está formado por cepas del linaje B1 de Barbosa *et al.* (2016)[12] y cepas del clado Ecuadorean de Peter *et al.* (2018)[10], SAM2 está formado por el linaje WB3 de Barbosa *et al.* (2016)[12] y diferentes cepas no asignadas de las Américas de Peter *et al.* (2018)[10], y SAM3 incluye el linaje B4 de Barbosa *et al.* (2016)[12], cepas de Cachaça de Barbosa *et al.* (2018)[13] y una cepa de destilado de Brasil de Gallone *et al.* (2016)[14].

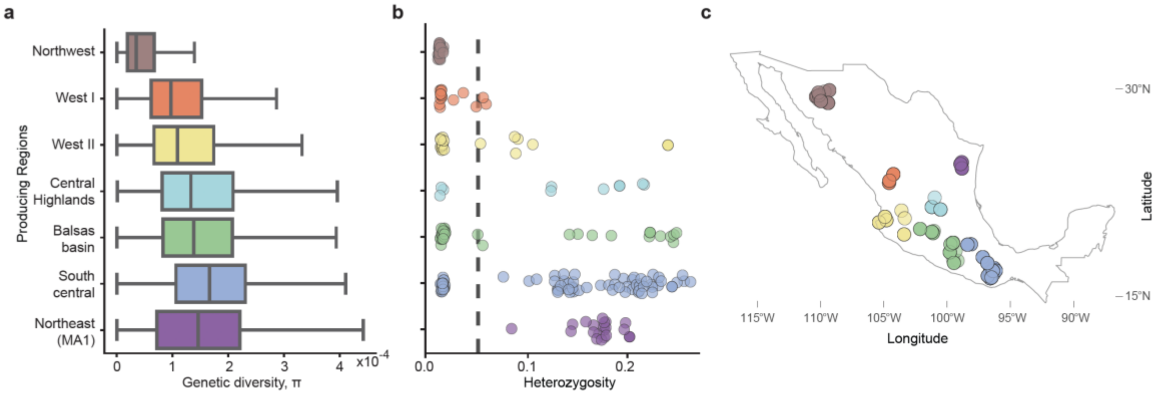

Figura Suplementaria S5. Las regiones bioculturales donde se fermenta agave para la producción de destilados muestran un gradiente de diversidad genética. A la izquierda, los diagramas de caja muestran los percentiles 25 y 75, con líneas verticales que indican las medianas de π calculadas en ventanas de 10kb. Centro, heterocigosidad a nivel genómico. La línea discontinua muestra el umbral que determina si las cepas son homocigotas según Peter *et al.*, (2018)[10]. Las regiones corresponden a lo descrito por Gallegos-Casillas *et al.*, (2024)[1].

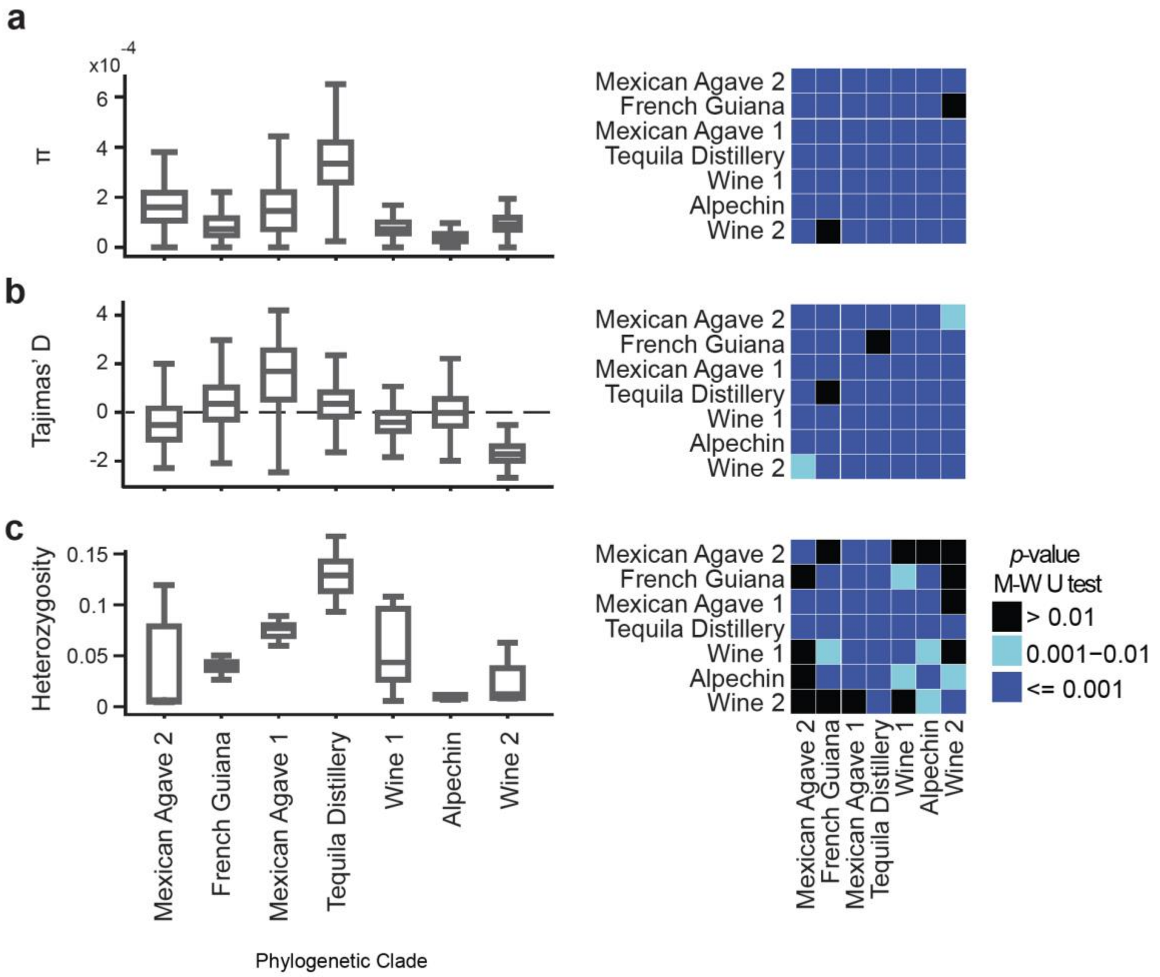

Figura Suplementaria S6. Parámetros de diversidad genética en clados de levaduras del grupo Neotropical. Los diagramas de caja representan los percentiles 25 y 75, con líneas horizontales que indican las medianas de diversidad genética π (**a**), *D* de Tajima (**b**) y heterocigosidad (**c**). Cada matriz a la derecha muestra el valor de *p* de una prueba pareada de U Mann-Whitney tal como se muestra en la leyenda. Se consideran clados con más de diez cepas disponibles (n=20 MA1; n=178 MA2; n=15 Tequila; n=29 FG; n=23 Wine1; Alpechin n=12; Wine2 n=11). Para los cálculos de diversidad genética y *D* de Tajima, el número de ventanas de 10kb usado para los diagramas de caja son MA1 n = 1,135, MA2 n = 1145, Tequila= 1,144, FG n = 1139, Wine1 n=1140, Alpechin n=1134 and Wine2 n=1137.

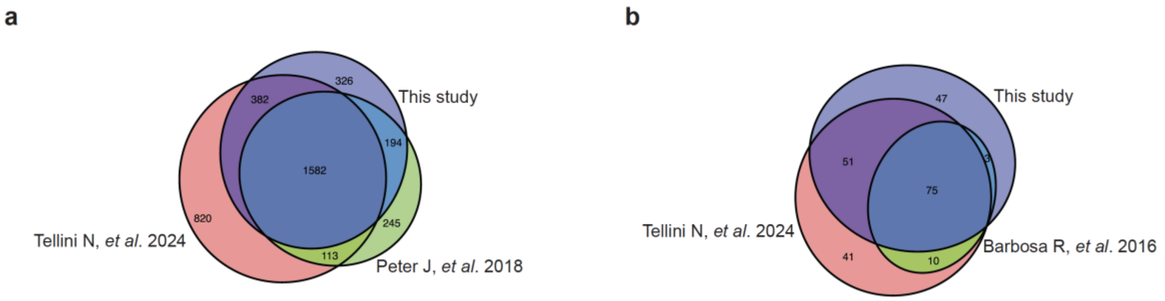

Figura Suplementaria S7. Comparación del método utilizado en este estudio para detectar genes introgresados con enfoques alternativos. **a** Diagrama de Venn que muestra el número de genes identificados como introgresados en 46 cepas compartidas utilizando tres métodos diferentes: este estudio; Tellini et al., 2024; Peter et al, 2018. **b**, Diagrama de Venn que muestra el número de genes identificados como introgresados en 3 cepas compartidas utilizando tres métodos de identificación diferentes: este estudio; Tellini et al., 2024; Barbosa et al, 2016.

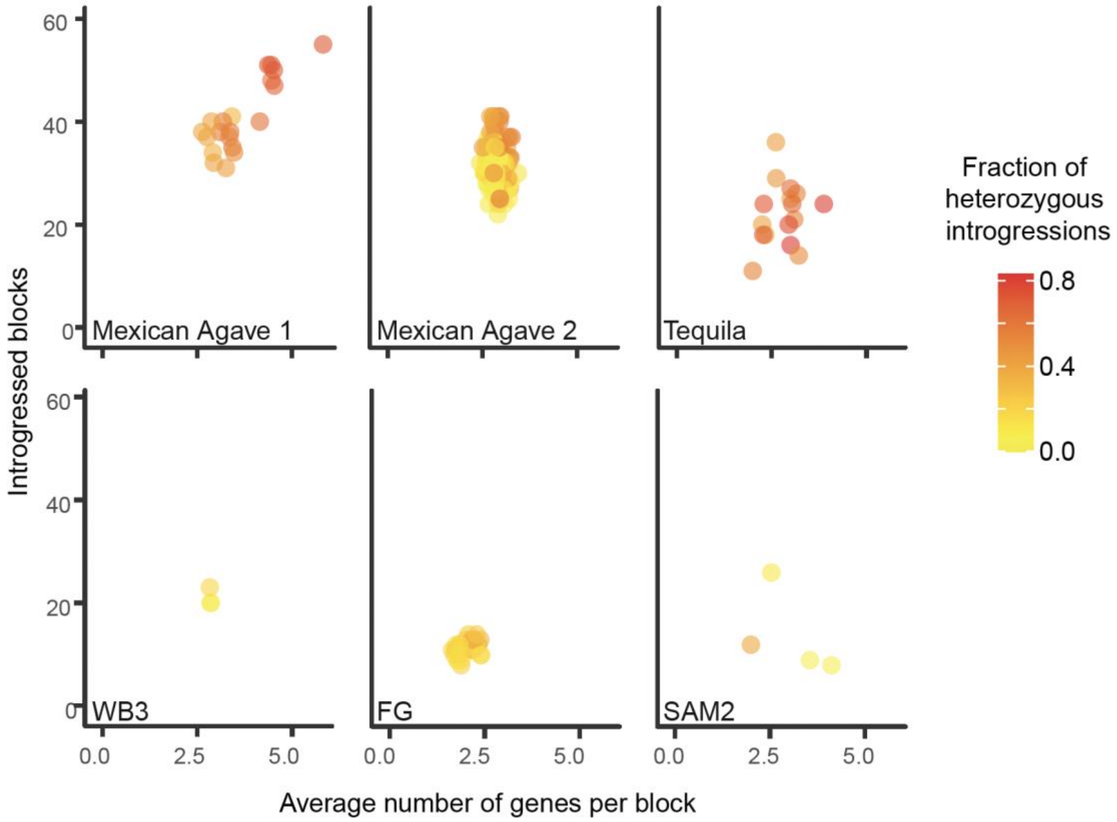

Figura Suplementaria S8. Tamaño del bloque de introgresión por clado del grupo Neotropical. Las cepas con más genes por bloque tienden a mostrar también un mayor número de bloques de introgresión. WB3, Wild Brazil 3; FG, French Guiana; SAM2, South American Mix 2.

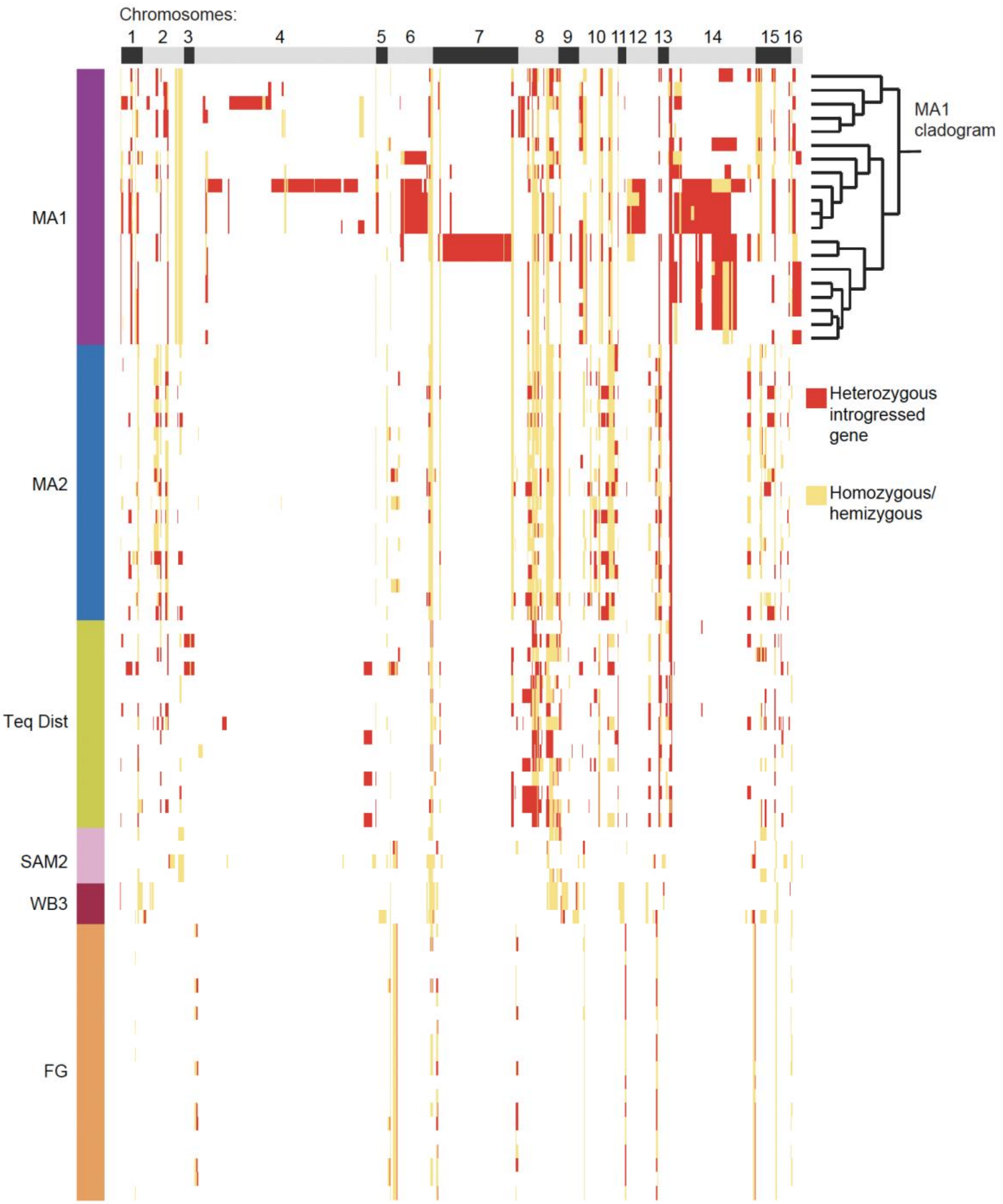

Figura Suplementaria S9. Cladograma de MA1 junto con la matriz de ausencia/presencia de genes introgresados por cepa. Las relaciones filogenéticas se extrajeron de la filogenia de la Figura 1B. El color de cada gen está determinado por la proporción del número de lecturas entre el alelo *S. cerevisiae* y el alelo de *S. paradoxus*. Las introgresiones con proporciones entre 0,25 y 4 se marcan como heterocigotas (rojo), mientras que las proporciones menores se marcan como homocigotas/hemicigotas. Para FG y MA2, se eligieron veinte secuencias aleatorias para ser representadas como referencia.

REFERENCIAS DE LAS FIGURAS SUPLEMENTARIAS

Las referencias de los pies de figuras suplementarias se encuentran numeradas conforme a la lista de Referencias del Material Suplementario de la versión original en inglés.

1. Gallegos-Casillas, P. *et al.* Yeast diversity in open agave fermentations across Mexico. *Yeast* **41**, 35–51 (2024).
2. López-Gallegos, C. *et al.* Ecological divergence of sympatric *Saccharomyces* species across wild and fermentative environments in the neotropics. 2025.05.31.656962 Preprint at https://doi.org/10.1101/2025.05.31.656962 (2025).
3. Lachance, M.-A. Yeast communities in a natural tequila fermentation. *Antonie van Leeuwenhoek* **68**, 151–160 (1995).
4. Verdugo Valdez, A. *et al.* Yeast communities associated with artisanal mezcal fermentations from *Agave salmiana*. *Antonie van Leeuwenhoek* **100**, 497–506 (2011).
5. Kirchmayr, M. R. *et al.* Impact of environmental conditions and process modifications on microbial diversity, fermentation efficiency and chemical profile during the fermentation of *Mezcal* in Oaxaca. *LWT - Food Science and Technology* **79**, 160–169 (2017).
6. Padilla-Camberos, E., Pinal-Zuazo, L. & Alvarez de la Cuadra Jacob, J. Catálogo de la colección de cultivos microbianos. (1994).
7. Kirchmayr, M. R. *et al. Manual para la estandarización de los procesos de producción del mezcal guerrerense*. vol. 1 (Centro de Investigación y Asistencia en Tecnología y Diseño del Estado de Jalisco A.C., Guadalajara, Jalisco. México, 2014).
8. Quezada, R., Gschaedler, A. & Kirchmayr, M. Characterization of microbial population dynamics associated with different juices of *Agave tequilana*. in *Sustainable and Integrated use of Agave* vol. 2016 2016 (2016, Zapopan, Jalisco, Mexico, 2016).
9. Álvarez-Ainza, M. L., Zamora-Quiñonez, K. A., Moreno-Ibarra, G. M. & Acedo-Félix, E. Genomic Diversity of *Saccharomyces cerevisiae* Yeasts Associated with Alcoholic Fermentation of Bacanora Produced by Artisanal Methods. *Appl Biochem Biotechnol* **175**, 2668–2676 (2015).
10. Peter, J. *et al.* Genome evolution across 1,011 *Saccharomyces cerevisiae* isolates. *Nature* **556**, 339–344 (2018).
11. Tellini, N. *et al.* Ancient and recent origins of shared polymorphisms in yeast. *Nat Ecol Evol* **8**, 761–776 (2024).
12. Barbosa, R. *et al.* Evidence of Natural Hybridization in Brazilian Wild Lineages of *Saccharomyces cerevisiae*. *Genome Biol Evol* **8**, 317–329 (2016).
13. Barbosa, R. *et al.* Multiple Rounds of Artificial Selection Promote Microbe Secondary Domestication—The Case of Cachaça Yeasts. *Genome Biology and Evolution* **10**, 1939–1955 (2018).
14. Gallone, B. *et al.* Domestication and Divergence of *Saccharomyces cerevisiae* Beer Yeasts. *Cell* **166**, 1397-1410.e16 (2016).
